# X chromosome variants are associated with male fertility traits in two bovine populations

**DOI:** 10.1186/s12711-020-00563-5

**Published:** 2020-08-12

**Authors:** Marina R. S. Fortes, Laercio R. Porto-Neto, Nana Satake, Loan T. Nguyen, Ana Claudia Freitas, Thaise P. Melo, Daiane Cristina Becker Scalez, Ben Hayes, Fernanda S. S. Raidan, Antonio Reverter, Gry B. Boe-Hansen

**Affiliations:** 1 School of Chemistry and Molecular Biosciences, The University of Queensland, Saint Lucia Campus, Brisbane, QLD 4072 Australia; 2 Queensland Alliance for Agriculture and Food Innovation (QAAFI), The University of Queensland, Saint Lucia Campus, Brisbane, QLD 4072 Australia; 3 CSIRO Agriculture and Food, Saint Lucia, QLD 4067 Australia; 4 School of Veterinary Science, The University of Queensland, Gatton Campus, Gatton, QLD 4343 Australia; 5 Faculty of Biotechnology, Vietnam National University of Agriculture, Hanoi, Vietnam; 6 Department of Animal Science, School of Agricultural and Veterinarian Science, São Paulo State University (UNESP), Jaboticabal, SP Brazil; 7 CSIRO Agriculture and Food, Hobart, TAS 7004 Australia

## Abstract

**Background:**

Twenty-five phenotypes were measured as indicators of bull fertility (1099 Brahman and 1719 Tropical Composite bulls). Measurements included sperm morphology, scrotal circumference, and sperm chromatin phenotypes such as DNA fragmentation and protamine deficiency. We estimated the heritability of these phenotypes and carried out genome-wide association studies (GWAS) within breed, using the bovine high-density chip, to detect quantitative trait loci (QTL).

**Results:**

Our analyses suggested that both sperm DNA fragmentation and sperm protamine deficiency are heritable (h^2^ from 0.10 to 0.22). To confirm these first estimates of heritability, further studies on sperm chromatin traits, with larger datasets are necessary. Our GWAS identified 12 QTL for bull fertility traits, based on at least five polymorphisms (P < 10^−8^) for each QTL. Five QTL were identified in Brahman and another seven in Tropical Composite bulls. Most of the significant polymorphisms detected in both breeds and nine of the 12 QTL were on chromosome X. The QTL were breed-specific, but for some traits, a closer inspection of the GWAS results revealed suggestive single nucleotide polymorphism (SNP) associations (P < 10^−7^) in both breeds. For example, the QTL for inhibin level in Braham could be relevant to Tropical Composites too (many polymorphisms reached P < 10^−7^ in the same region). The QTL for sperm midpiece morphological abnormalities on chromosome X (QTL peak at 4.92 Mb, P < 10^−17^) is an example of a breed-specific QTL, supported by 143 significant SNPs (P < 10^−8^) in Brahman, but absent in Tropical Composites. Our GWAS results add evidence to the mammalian specialization of the X chromosome, which during evolution has accumulated genes linked to spermatogenesis. Some of the polymorphisms on chromosome X were associated to more than one genetically correlated trait (correlations ranged from 0.33 to 0.51). Correlations and shared polymorphism associations support the hypothesis that these phenotypes share the same underlying cause, i.e. defective spermatogenesis.

**Conclusions:**

Genetic improvement for bull fertility is possible through genomic selection, which is likely more accurate if the QTL on chromosome X are considered in the predictions. Polymorphisms associated with male fertility accumulate on this chromosome in cattle, as in humans and mice, suggesting its specialization.

## Background

Male fertility is of interest to and concerns two distinct fields: clinical medicine, which aims at identifying and treating subfertility, and livestock breeding, which relies on optimal fertility rates for efficient production. Male fertility indicators, such as sperm susceptibility to DNA fragmentation, sperm morphology, or testicular size, are used in a clinical context and, also, to evaluate the breeding capacity of livestock species [1–3]. Fertility indicators are often heritable and represent complex phenotypes that are controlled by multiple genes [4, 5]. In our study, we used 25 phenotypes as indicators of bull fertility in genome-wide association studies (GWAS) that were performed in two breeds. These 25 phenotypes were categorized into four groups: traditional semen quality assessment (including sperm morphology, concentration, and motility), the level of inhibin hormone and scrotal circumference (SC), sperm chromatin susceptibility to fragmentation, and sperm protamine deficiency. Together, these phenotypes offer a comprehensive dataset to investigate the underlying genetics of bull fertility.

The first group of phenotypes represents standard measurements in evaluations of bull-breeding soundness, including sperm morphological abnormalities. Indeed, sperm morphology, concentration, and motility are routinely measured [6], and visual scores of colour, density, and sperm mass activity are also used in the field to provide initial clues of semen concentration and sperm motility. Visual scores are subjective and influenced by human error, as are the count of sperm morphological defects and sperm concentration using microscopy, but to a lesser extent. Morphology evaluation categorizes sperm morphological abnormalities in subcategories such as head (HA), midpiece (MA) and tail abnormalities (TA). A common abnormality in the bovine sperm is the retention of cytoplasmic droplets that can be proximal or distal to the midpiece. Within this first group of routine phenotypes, the percentage of sperm with normal morphology is considered the best indicator of bull fertility [3, 7].

The second group of phenotypes consists of measurements of inhibin hormone levels and of SC. Levels of inhibin at 4 months of age and SC, which is measured between 12 and 24 months of age, are indicators of pubertal development [8, 9]. Puberty affects semen quality and eventually leads to sexual maturity [10]. Veterinarians and researchers often use phenotypes of the first and second group to determine if a bull is mature in terms of fertility [10]. Previous GWAS focussed only on these two first groups of phenotypes [4, 5].

The third and fourth groups of phenotypes correspond to measurements that are associated with the chromatin structure of sperm nuclei. Two flow cytometric methods—the sperm chromatin structure assay (SCSA) [1], and the sperm protamine deficiency assay (SPDA) [11]—measure these clinically relevant phenotypes by evaluating thousands of spermatozoa. SCSA measures sperm chromatin susceptibility to fragmentation as an index, known as the DNA fragmentation index (DFI). This index has been associated with lower fertility and miscarriages in humans [2, 12], smaller litter size in pigs [13], and reduced success of artificial insemination in bulls [14]. Higher DFI values result in embryonic development failure [15]. The clinical value of SCSA is evident, but the cause for increased DFI (genetic or otherwise) is largely unknown and likely multifactorial [16, 17]. The proposed causes of increased DFI include environment stressors, genetics, compromised spermatogenesis, or inappropriate sperm chromatin structure, which could be due to a deficiency in protamine [18, 19]. Protamines replace histones during spermatogenesis and are required to form and stabilize the highly condensed structure of sperm chromatin [20–22]. Sperm chromatin susceptibility to fragmentation is associated with protamine deficiency in both bulls and humans [11, 23]. In this study, we take a further step and investigate the genomic regions associated with both sperm chromatin fragmentation and sperm protamine deficiency, their heritability and the genetic correlation between these phenotypes.

SCSA simultaneously determines DFI and the percentage of sperm with high DNA stainability (HDS). Whereas DFI is related to DNA breaks (single-strain DNA), HDS may be related to the retention of nuclear histones, which is consistent with immature spermatozoa [16, 24]. Genome-wide histone retention in spermatozoa may be a sign of immaturity, but it is hypothesized that specific retention of some histones is highly important shortly after fertilization. Histone retention creates the possibility of paternal gene expression, correlates with developmental regulators, and contributes to early embryonic development in humans [25, 26]. Hence, in our study, we investigated the inheritance of sperm chromatin phenotypes (such as DFI, HDS, and protamine deficiency) as a proxy for histone-retention inheritance, which is poorly understood in bulls. Furthermore, sperm chromatin phenotypes are related to paternal epigenetic markers, such as posttranslational modifications of retained histones in humans [27]. Immature spermatozoa, with retained histones, may also present a higher proportion of proximal cytoplasmic droplets, the common morphological defect [28, 29]. Proximal cytoplasmic droplets are considered an indicator of sperm immaturity in bulls and have been shown to be associated with sperm DNA fragmentation [29]. Droplets with cytoplasmic content may increase the generation of reactive oxygen species, and this contributes to sperm DNA fragmentation [30]. Phenotypic correlations between an increased percentage of sperm with proximal cytoplasmic droplets and DFI have been reported in both humans and bulls [29, 31, 32]. Here, we investigate the genetic correlations between these phenotypes.

After performing a GWAS for each phenotype in each breed, we applied a QTL detection method adapted from van den Berg et al. [33], which is based on the relative significance of neighbouring single nucleotide polymorphisms (SNPs). In short, our objectives were to estimate the heritability of bull-fertility phenotypes, their genetic correlations, and identify QTL.

## Methods

### Animals

Institutional Animal Care and Use Committee approval was not required for this study because the data and samples used were obtained from existing databases and storage banks. Animals were bred by the Cooperative Research Centre for Beef Genetic Technologies (Beef CRC), and details on these populations were published prior to this study [34]. In brief, 1099 Brahman bulls and 1719 Tropical Composites were used, but the exact number of records for each trait varied (see Table 1). Brahman are animals that are mostly of *Bos indicus* origin, while Tropical Composites are cattle that originate from planned crossbreeding between *Bos indicus* and *Bos taurus* breeds. The genomic evidence for the breed composition of these Australian cattle was discussed in previous studies [35, 36]. Breed composition can affect GWAS and so we favour within-breed analyses.

**Table 1:** Male fertility phenotypes: brief description and summary of genome-wide association results

Phenotypes	Brief description	Brahman					Tropical Composites				
		N	Mean	SD	P < 10^−8^	h^2^ (SE)	N	Mean	SD	P < 10^−8^	h^2^ (SE)
Group 1											
PNS	% normal sperm	1023	0.70	0.22	166	0.35 (0.07)	1648	0.72	0.19	4	0.29 (0.05)
PD	% sperm with proximal droplets	1023	0.07	0.15	0	0.35 (0.07)	1648	0.04	0.07	1593	0.21 (0.04)
DD	% sperm with distal droplets	1023	0.03	0.05	1	0.10 (0.05)	1648	0.03	0.05	0	0.13 (0.04)
TD	% sperm with droplets (total)	1023	0.10	0.16	0	0.33 (0.07)	1648	0.07	0.09	591	0.18 (0.04)
HA	% sperm with head abnormalities	1023	0.09	0.11	821	0.27 (0.06)	1648	0.10	0.14	0	0.30 (0.05)
MA	% sperm with midpiece abnormalities	1023	0.10	0.11	487	0.07 (0.05)	1648	0.10	0.10	0	0.21 (0.05)
TA	% sperm with abnormal tail	1023	0.00	0.01	0	0.00 (0.03)	1648	0.00	0.01	0	0.00 (0.02)
COL	Colour, visual score 1–5	1099	3.18	0.95	0	0.09 (0.05)	1719	3.38	0.94	0	0.05 (0.03)
MOT	% progressive sperm motility	1099	69.29	23.61	0	0.00 (0.04)	1719	72.09	22.15	0	0.13 (0.04)
MAS	Mass activity, visual score 1–5	1099	2.59	1.09	0	0.09 (0.05)	1719	2.81	0.98	0	0.12 (0.04)
CON	Sperm concentration, ×10^6^/ml	592	266.11	287.41	0	0.03 (0.06)	538	237.18	238.23	0	0.04 (0.07)
DEN	Density, visual score 1–5	1,098	3.07	0.90	0	0.13 (0.06)	1716	3.21	0.84	0	0.08 (0.03)
Group 2											
Inhibin	Blood levels of inhibin at 4 months, (ng/ml)	806	7.41	1.89	38	0.63 (0.08)	1329	7.76	1.88	1	0.56 (0.06)
SC12	SC at 12 months, cm	1098	21.40	2.41	3	0.57 (0.06)	1717	25.86	3.15	0	0.65 (0.04)
SC18	SC at 18 months, cm	1098	26.70	2.71	0	0.61 (0.06)	1719	29.82	2.82	2	0.67 (0.04)
SC24	SC at 24 months, cm	1098	29.89	2.86	1	0.63 (0.06)	1719	31.42	2.80	49	0.70 (0.04)
Group 3											
PIC3	Sperm with intact chromatin in FL3, %	585	89.85	8.71	0	0.12 (0.08)	511	89.27	6.89	0	0.16 (0.09)
DFI3	DNA fragmentation index in FL3, %	585	3.77	4.23	5	0.10 (0.07)	511	4.47	4.73	0	0.21 (0.10)
HDS3	High DNA stainability in FL3, %	585	6.38	6.76	0	0.05 (0.07)	511	6.27	3.96	0	0.20 (0.11)
PIC4	Sperm with intact chromatin in FL4, %	585	86.95	10.26	0	0.17 (0.08)	511	85.32	11.28	0	0.17 (0.09)
DFI4	DNA fragmentation index in FL4, %	585	6.41	7.13	8	0.15 (0.09)	511	8.38	10.38	0	0.15 (0.09)
HDS4	High DNA stainability in FL4, %	585	6.64	7.23	0	0.04 (0.07)	511	6.29	3.94	0	0.20 (0.11)
Group 4											
LCB	Low CMA3 binding (intact protamination), %	592	84.50	14.78	0	0.22 (0.09)	538	87.97	11.27	0	0.13 (0.08)
MCB	Medium CMA3 binding (medium protamination), %	592	14.86	14.42	11	0.17 (0.08)	538	12	11.25	0	0.13 (0.09)
HCB	High CMA3 binding (protamine deficiency), %	592	5.99	10.68	0	0.01 (0.06)	538	5.44	6.06	0	0.07 (0.08)

N, number of SNPs associated (P < 10^−8^); Mean, mean phenotypes; SD, standard deviation of mean phenotypes; h^2^, SNP-derived heritability; SE, standard error of h^2^

The number of records were smaller for sperm chromatin phenotypes: at most, 592 for Brahman and 538 for Tropical Composites. Sample size clearly influences the power of any GWAS and we calculated the power for each analysis by using the online tool developed by Visscher and his team [37]. Their method uses the estimated heritability, the sample size and the empirical variance of the genetic relationships estimated in the genomic relationship matrix (GRM) to estimate the power of GWAS. It should be noted that this method for estimating power was developed for large populations of unrelated individuals i.e. humans, which are different from our cattle populations that include a large number of half-siblings. Estimated power values for each phenotype in each breed are in Additional file 1: Table S1. For some traits with lower heritability and/or smaller sample sizes, the analyses of power indicate that larger datasets will be needed to identify/confirm QTL for these traits.

### Phenotypes

Classic indicators of bull fertility used as phenotypes are traits derived from sperm morphology assessments of 100 sperm cells under light microscopy (i.e., all sperm morphological abnormalities), SC measured in cm with a standard measuring tape, and the level of the inhibin serum hormone measured at 4 months, as described in the initial Beef CRC project design [34]. We also added two assays that measure sperm chromatin phenotypes: the sperm protamine deficiency assay (SPDA) and the sperm chromatin structure assay (SCSA).

#### Sperm protamine deficiency assay

The percentages of sperm cells with high-, medium-, and low-protamine content (HPC, MPC and LPC, respectively) were estimated using the SPDA methodology described in previous studies [11, 29, 32]. Briefly, sperm protamine was assessed using a fluorochrome that competes for protamine-binding sites at the minor groove of a DNA strand [38, 39]. Thawed sperm samples were analysed using the Beckman Coulter Gallios flow cytometer, and the Kaluza software (version 1.1) was used to generate the phenotypes for genetic analyses (see Additional file 2).

#### Sperm chromatin structure assay

Samples thawed for SPDA were also used for SCSA, which measured three related phenotypes: percentage of sperm with intact chromatin (PIC), DFI, and HDS. SCSA was conducted according to the protocol described by Evenson and Jost [40], which uses the metachromatic properties of acridine orange to assess DNA fragmentation. For every six-test samples, a reference sample was analysed to ensure stability of the instrument using the same flow cytometer and analysis software equipment as described above (see Additional file 2). Two values for PIC, i.e. DFI and HDS were determined using FL3 and FL4 fluorescence, which resulted in two phenotypes for each measurement of sperm chromatin staining [29, 32].

### Genotypes and imputation

The Illumina *AB* format was used for genotype calling and then genotypes were coded as 0, 1 or 2 with reference to the number of *B* alleles carried by each animal, at each locus. Genotyping was done with a medium-density chip (54,000 SNPs) for most of the bulls with fertility phenotypes, as reported in the initial GWAS [4, 5]. In addition, 999 bulls from the same populations were genotyped in the current study to expand the GWAS to the sperm chromatin phenotypes. These additional genotypes were acquired with the GeneSeek Genomic Profiler chip (also Illumina Infinium chemistry), which features approximately 78,000 SNPs. Duplicated samples were included in both chip assays for quality control. Animals with a call rate lower than 90% were discarded. The SNPs that mapped to more than one position on the genome or had a call rate lower than 90% were discarded. For some SNPs, we observed bulls that were homozygous *AA* and bulls that were homozygous *BB*, but no *AB* heterozygous bull was observed, and so we discarded these SNPs (as these observations point to a possible genotyping error) (except for SNPs on chromosome X). The genotyping results for the “50 K” chip were reported in the initial GWAS, in which 50,353 and 48,821 SNPs passed control quality in Brahman [4] and Tropical Composites [5], respectively. After genotyping with the “70 K” chip, 68,406 SNPs were available after quality control for 999 bulls.

A panel of 1174 cattle were genotyped with the BovineHD Illumina chip (~ 770,000 SNPs) and used as a reference for imputation of lower density genotypes (“50 K” and “70 K”) up to a higher density. The reference panel included the sires of most of the animals that were genotyped with the lower density panels, some of the dams and additional randomly selected *Bos indicus* and composite cattle of five breeds (Brahman n = 519, Belmont Red n = 97, Droughtmaster n = 45, Santa Gertrudis n = 168, and Tropical Composite n = 351). This panel of breeds was part of a larger experiment with 10,181 animals, which allows for accurate imputation of tropical cattle as described before [41]. For this study, we remapped all the original SNP genotypes to the new bovine reference genome (ARS_UCD1.2, GenBank assembly accession GCA_002263795.2) [42], before performing a new imputation.

Before imputation, all SNP genotypes were phased using the Eagle software [43] to complete the sporadic missing genotypes in the high-density reference panel and not the missing genotypes in the lower-density datasets. Then, the lower-density genotypes were imputed using Minimac3 for all autosomes [44] and Minimac4 for chromosome X. Chromosome X was imputed after separation of the pseudoautosomal and non-pseudoautosomal regions, based on a recent definition of the boundaries between these regions [45]. After imputation, SNPs with an imputation accuracy r^2^ higher than 0.8 were retained, resulting in a final dataset of 722,208 SNPs with genotypes available for the studied bulls, in both breeds.

### Genomic relationship matrix (GRM)

To perform the GWAS, we built a GRM for each breed using all the SNPs that had a MAF higher than 0.05 (within-breed). Each GRM was built using the first method proposed by VanRaden [46]. We used all the default parameters in the SNP & Variation Suite (SVS) software (release 8.3.0, Golden Helix), including the overall normalization method, as described by Taylor [47]. The GRM was corrected for sex using the full dosage compensation method. The estimated relationships for both breeds (the GRM off-diagonal elements) had a variance of ~ 0.002 (see Additional file 2). These relationships estimated from genotypes conform with the expectations of having measured the progeny of 55 Brahman sires and 56 Tropical Composite sires [48]. We used these precomputed GRM to fit the random polygenic effect in all our models.

### Genome-wide association studies

After the GRM was computed, SNPs with a low MAF (lower than 0.05) were included again in the single-SNP association tests, to accommodate breed differences and to facilitate GWAS comparisons. MAF were reported within-breed together with the association results (see Additional files 3, 4: Tables S2, S3) to provide an informed interpretation of the reported SNP effects.

GWAS were performed by using an additive mixed model to compute single-trait-single-SNP associations. All available 722,208 SNPs were tested individually for each phenotype, in each breed. Within-breed GWAS allowed the detection of breed-specific QTL, which were expected for these populations [4, 5] (see Additional file 2, and Additional file 5: Figures S1 to S11). The GWAS models used the precomputed GRM, contemporary groups as fixed effects and age as a covariant. The effects of contemporary groups and age for the measured traits had been examined in previous studies [4, 5, 49–51]. Contemporary groups were defined as cohorts of bulls that were born in the same year and raised together in the same location. SNP additive effects for each trait were calculated by fitting these mixed models in the SVS software (release 8.3.0, Golden Helix).

The precomputed GRM (including autosomes and X-linked SNPs) was also used to estimate genetic correlations and heritability for the studied phenotypes, within-breed. SNP-derived heritabilities and their standard errors were estimated by fitting the above-mentioned mixed models in the SVS software (release 8.3.0, Golden Helix). Genetic correlations were estimated by fitting bivariate genomic mixed models using the Qxpak5 software [52]. Fixed effects and age were included in the models, as described above.

The significant SNPs reported for each trait followed the thresholds that we defined: a P-value lower than 10^−8^ and a MAF higher than 0.05 (within breed), which is a conservative Bonferroni correction, because it considered all SNPs as independent tests (P = 6.8 × 10^−8^ equivalent to P = 0.05 when considering all the tested SNPs).

### Boundaries of quantitative trait loci

Given the large number of significant SNPs present in the large genomic regions, especially on the X chromosome, it was important to identify QTL boundaries by applying a methodology adapted from van den Berg et al. [33]. First, we identified each QTL peak by ranking the SNPs according to their P-values (lower to higher). Only SNPs with a P lower than 10^−8^ were considered as peak SNPs and used to define QTL boundaries. After selecting the most significant SNP on each chromosome, we tested the other significant SNPs within 0.5 Mb on either side of the peak SNP to determine if they fitted the following criteria: –log10 (P) ≥ -log10 (Ppeak) × 2/3. When a SNP did fit the criteria, it was considered as part of the same QTL. The borders of each QTL were expanded iteratively (one 0.5-Mb window at a time) until no more SNPs satisfied the condition of − log10 (P) ≥ − log10 (P_peak_) × 2/3. When no further SNPs fitted the criteria, the next QTL started in a region 0.5 Mb away from the previous QTL. In the case of a new QTL, a new peak was set until there were no SNPs with a P value lower than 10^−8^ to call new peaks. As an additional condition, a minimum of five SNPs was required to define a QTL. If there were less than five SNPs, the QTL was not reported, as in van den Berg’s work [33].

## Results

### Heritability and genetic correlations

In this study, we targeted 25 phenotypes that were measured as indicators of bull fertility. The SNP-derived heritability of phenotypes ranged from very low (i.e., 0.00 for tail abnormalities) to high (i.e., 0.56 to 0.70 for SC measurements, see Table 1). Sperm chromatin fragmentation and sperm protamine deficiency had heritability estimates that ranged from 0.10 to 0.22, which is similar to that of many fertility traits. We also report the standard errors for these SNP-derived heritabilities, which ranged from 0.02 to 0.11. A word of caution is needed regarding some traits for which the number of available animals was smaller, such as sperm chromatin phenotypes (~ 500 bulls). For traits with low heritability estimates and a smaller number of animals, the power to detect the genetic variance explained by all SNPs was limited in our study. However, for many traits, we were still able to estimate with confidence their genetic variance (see Additional file 1: Table S1), which is probably due to large half siblings families being recorded [5, 48]. The methods used to calculate power estimates were developed for human populations with unrelated individuals [37]. We decided to set a stricter value (α = 0.001) then the default value (α = 0.05) for type one error in these calculations, because it is the first time that these methods are applied to livestock data.

Given the size of the samples and power considerations, in this paper, we report only the most significant SNP associations based on a strict Bonferroni correction (see Table 1). In addition, we present only the QTL that had at least five significant SNP associations for a given trait (see Table 2).

**Table 2:** Boundaries of the identified QTL for bull fertility phenotypes

Trait	Chr.	Start	End	Peak position	P-value	MAF of peak SNP	Nearest gene
QTL in Brahman							
PNS	X	45,230,870	49,785,424	45,230,870	4 × 10^−10^	0.12	*DIAPH2*
HA	X	47,584,977	52,590,608	49,961,998	7 × 10^−15^	0.10	*GPRASP1*
MA	X	4,072,268	7,370,241	4,929,592	1 × 10^−17^	0.17	*ATP1B4*
Inhibin	2	107,523,103	107,631,932	107,558,848	4 × 10^−18^	0.22	*SLC4A3*
DFI3	11	98,399,847	98,412,314	98,412,314	2 × 10^−10^	0.06	*TOR2A*
QTL in tropical composites							
PD	X	1,380,454	8,539,246	4,280,429	2 × 10^−20^	0.09	*NFKB*
	X	29,691,394	38,007,510	33,595,924	7 × 10^−13^	0.13	*MAMLD1*
	X	45,016,203	47,360,189	45,984,909	2 × 10^−12^	0.17	*DIAPH2*
	X	55,280,920	55,317,351	55,297,857	2 × 10^−10^	0.15	*RNF128*
TD	X	1,380,454	8,539,246	5,091,253	2 × 10^−18^	0.13	*CUL4B*
	X	40,278,180	41,069,102	40,509,046	2 × 10^−9^	0.17	*PCDH11X*
SC24	5	45,743,023	49,417,084	48,275,554	8 × 10^−14^	0.24	*MSRB3*

QTL, quantitative trait loci identified for each phenotype, see Table 1 for phenotype description; Chr, chromosome to which the QTL maps; Start, position (bp) on the chromosome where the QTL starts; End, position (bp) on the chromosome where the QTL ends; Peak position, position (bp) on the chromosome where the peak SNP is localized; peak SNP is the marker with the most significant association in terms of P-value for each QTL; the nearest gene is reported based on the peak SNP position; MAF, minor allele frequency for the SNP at the peak position, in the breed that is relevant to the QTL

**Fig. 1:**
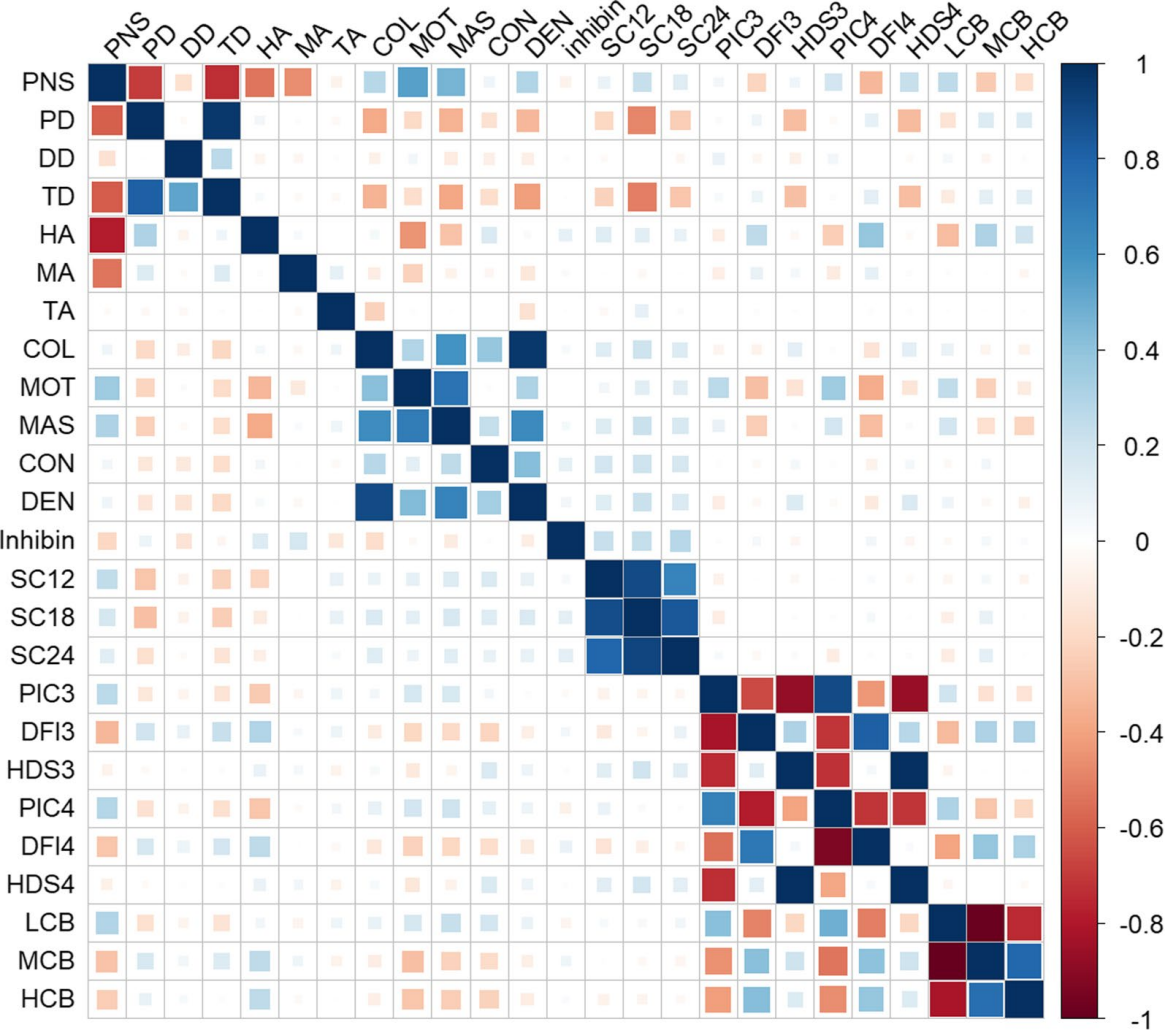
Genetic correlations for 25 bull fertility phenotypes in Brahman (above the diagonal) and Tropical Composites (below the diagonal). Negative genetic correlations are in red and positive correlations are in blue. Larger squares indicate more extreme correlations (closer to 1 or − 1); (see Additional file 5: Figure S1) for the numerical values corresponding to each estimated pairwise correlation

The estimated genetic correlations across the 25 phenotypes followed our expectations, given the current knowledge on these traits, and are presented in Fig. 1 and also (see Additional file 5: Figure S1). As expected, the correlations between common morphological sperm abnormalities and the percentage of normal sperm (PNS) were negative. Positive correlations between colour, motility, mass activity, concentration, and density were observed. The three SC measurements were highly correlated for both breeds, as were the indicators of sperm chromatin configuration with each other. Such correlations were expected because all the sperm chromatin phenotypes were derived by using the same method. Similarly, sperm protamine phenotypes were highly correlated in the expected directions. Sperm protamine deficiency was correlated with sperm DNA fragmentation. Proximal cytoplasmic droplets were correlated with HDS3 and HDS4 in Tropical Composites, but not in Brahman. The observed genetic correlations agree with the grouping of phenotypes as shown in Table 1.

### Genome-wide association studies within each breed for each phenotype

**Fig. 2:**
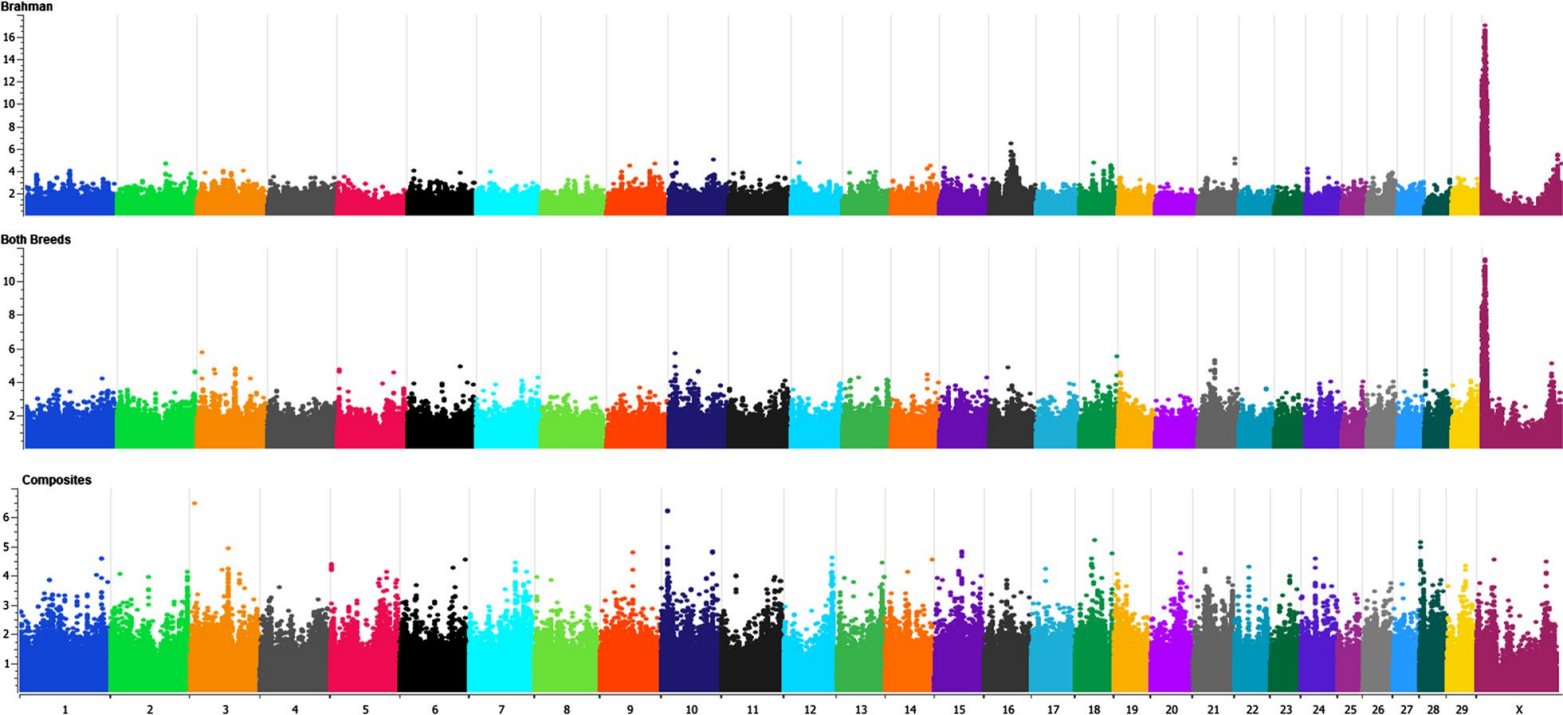
Genome-wide association studies performed in Brahman only (top), in both breeds together (middle) and in Tropical Composites only (bottom) for sperm mid-piece morphological abnormalities (MA). Significant SNP associations for the percentage of sperm with MA were identified only in Brahman. The significance of the associations decreased when the two breeds were analysed together (fitting breed as a fixed effect in the model). We focused on within-breed GWAS to be able to identify associations, and QTL that are breed-specific

The GWAS were performed by fitting an animal model with full dosage compensation for SNPs on the X chromosome. Each SNP was tested for their additive effect on each trait, within breed. Across-breed GWAS can leave undetected results that are significant when analysing breeds separately, as observed for sperm mid-piece abnormalities (Fig. 2). Thus, our study focused on within-breed analyses to avoid such issues.

Among the significant SNPs for each breed, most of them mapped to chromosome X. The remaining significant SNPs identified for the Brahman breed mapped to either chromosome 11 or chromosome 2. In Tropical Composites, apart from the associations on the X chromosome, some significant SNPs for scrotal circumference mapped to chromosome 5. The complete GWAS results with MAF, P-values, allele substitution effects (ASE), their standard errors (SE) and the percentage of the genetic variance explained per SNP are in Table S2 for Brahman and Table S3 for Tropical Composites (see Additional files 3, 4: Tables S2, S3).

### Boundaries of the quantitative trait loci

**Fig. 3:**
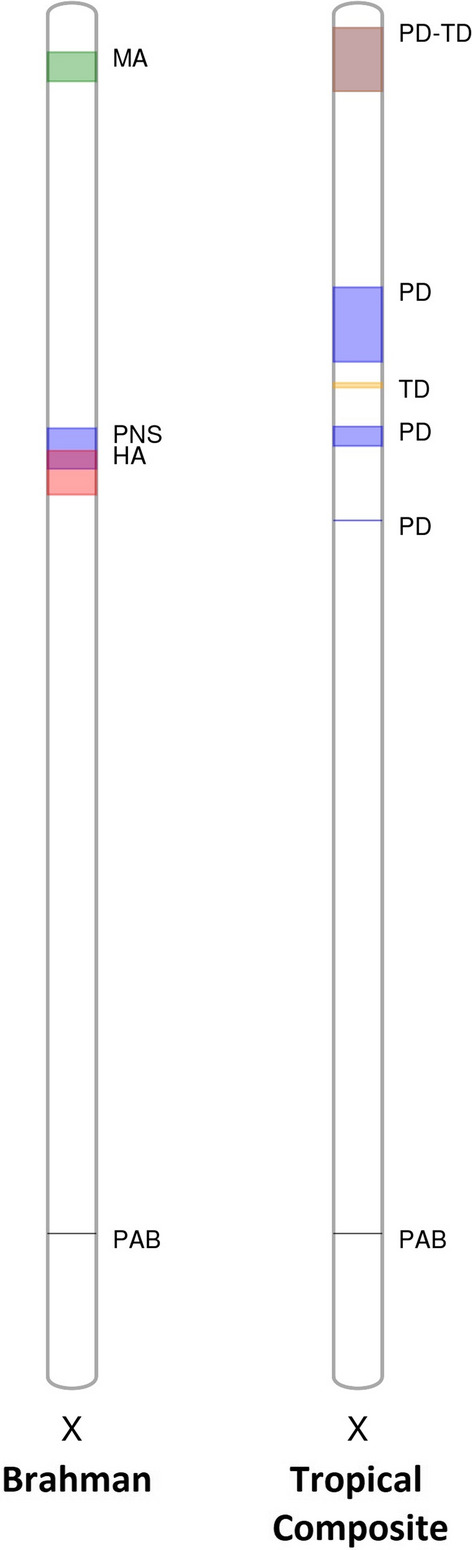
Association results on the X chromosome of Brahman (left) and Tropical Composite bulls (right). Each coloured region represents a QTL, defined by at least five SNPs associated (P < 10^−8^) with each phenotype. Phenotype abbreviations are provided next to each QTL (phenotypes and abbreviations, as described in Table 1). The X chromosome harboured QTL in both breeds; some QTL overlap. The pseudo-autosomal region boundary (PAB) is marked on the chromosome to show that all reported QTL were localized before the boundary, in the non-autosomal region

The proposed QTL are summarized in Table 2. The associations that were detected on chromosome 2 for inhibin level and chromosome 11 for DFI3 in Brahman and chromosome 5 for SC24 in Tropical Composites suggest QTL that span a “narrow” interval, when compared to some “broad” QTL identified on the X chromosome for sperm morphology traits. Evidence for QTL in both breeds was found only on the X chromosome, see Fig. 3.

The boundaries of each reported QTL are informative for the identification of genomic regions that may be pleiotropic (Table 2). For example, QTL for PNS overlapped with QTL for HA in Brahman and PD in Tropical Composites. Some QTL identified on the X chromosome span a few Mb. As a step towards the fine-mapping of these QTL regions, it is possible to focus first on the regions that are shared across breeds and traits. Considering both breeds, we suggest two consensus regions for sperm morphological abnormalities. The first consensus region starts at 4,072,268 bp and ends at 7,370,241 bp, as the boundaries for the Brahman QTL for MA are within the larger QTL for PD and TD in Tropical Composites. These QTL regions encompass many genes. The second consensus region is an overlapping region between the Brahman QTL for PNS and a QTL for PD in Tropical Composites; it starts at 45,230,870 bp and ends at 47,360,189 bp.

In Brahman, all significant SNPs (P < 10^−8^) that mapped to chromosome 2 were associated with inhibin levels. Suggestive SNP associations clustered on chromosomes 2, 3 and 5 for inhibin levels in Tropical Composites (P < 10^−7^) (see Additional file 5).

In Brahman, the associations on chromosome 11 suggest a QTL that is relevant for sperm chromatin fragmentation at 98.4 Mb. In this region, BovineHD1100028635 is the most significant SNP associated with DFI3 in Brahman (P < 10^−8^) and the nearest gene is *TORA2*. We used non-additive models to verify if any recessive or dominant effects were present for SNPs in all QTL identified. The only instance when the non-additive models could better capture SNP associations was for chromosome 11 and the DFI3 phenotype in Brahman. The recessive model for DFI3, resulted in extreme SNP associations (P = 3.38 × 10^−30^), compared to the result with the dominant (P = 2.74 × 10^−10^) and the additive models (P = 2.46 × 10^−10^). Thus, recessive inheritance may be considered when searching for the causal variant that underlies DFI3 variance in Brahman.

SNP *rs452801659* (X:79,745,560) was previously genotyped as part of a study on the candidate gene *TEX11*, and was found to be associated with scrotal circumference traits in a sub-set of our bull populations [53]. Thus, although this SNP is not included in any of the QTL defined here, we tested its association with the new traits under investigation (i.e. the sperm chromatin traits measured by SCSA and SPDA). In Brahman, *rs452801659* was not associated with sperm chromatin fragmentation, but suggestive associations were detected with sperm protamine deficiency traits: LCB (P = 1.29 × 10^−5^), MCB (P = 3.26 × 10^−6^), and HCB (P = 1.58 × 10^−4^). In Tropical Composites, no associations were observed between *rs452801659* and the new sperm chromatin or protamine traits. We confirmed the already known association between this SNP and scrotal circumference with the following results: P = 2.00 × 10^−6^ (SC12), P = 8.65 × 10^−7^ (SC18), and P = 1.70 × 10^−6^ (SC24) for Tropical Composites, and P = 1.20 × 10^−3^ (SC12), P = 6.64 × 10^−6^ (SC18), and P = 2.30 × 10^−5^ (SC24) for Brahman. These SNP associations indicate that the stringent conditions used here to define the 12 detected QTL ensure reliable associations, but probably fail to capture all the genomic regions that are relevant to bull fertility.

## Discussion

Estimates of heritability and genetic correlations guide the choice of phenotypes for selective breeding. Genetically-correlated phenotypes with a high heritability can increase the rate of genetic gain and lift bull fertility. Our results confirm that PNS and SC are useful indicators of bull fertility and suggest that there is value in adding sperm chromatin and sperm protamine measurements to evaluate bulls, especially for the high-value animals used for artificial insemination. Sperm chromatin and sperm protamine phenotypes are moderately heritable, approximately 20% for DFI in Tropical Composites and for LCB in Brahman. Studies on twins in humans [54] and in Holstein bulls [55] corroborate that sperm chromatin phenotypes are heritable. The study in humans investigated 300 men and proposed heritability estimates of 68% and 72% for SCSA measurements [37]. A microscopy-based method for measuring sperm chromatin fragmentation, performed on 201 Holstein bulls, resulted in estimates of heritability close to 41% [38]. In short, our results and previous studies confirm that sperm chromatin phenotypes are heritable, and thus, useful indicators of bull fertility for selective breeding. Moreover, correlations between these sperm chromatin traits and the other traits are favourable. For example, DFI3 is negatively correlated with PNS (r^2^ ~ 0.23–0.33), which means that bulls with a higher PNS will have less DNA fragmentation, and will thus be more fertile according to both indicators.

Given the genetic correlations between phenotypes of different groups, it is possible to consider that sperm chromatin phenotypes can be combined with PNS and SC, to form a comprehensive male fertility index. Selection indices that include fertility traits are currently implemented in the dairy industry [56] and these could be recommended for beef cattle too. An index that benefits from the favourable genetic correlations between traits that describe different aspects of bull fertility is a sensible approach to selective breeding. The clinical use of complementary indicators for male fertility is largely accepted, when deciding on bull fitness for the mating season [6, 10, 32]. We propose to extend this rationale to create a multi-trait index that captures genetic merit for bull fertility.

The genetic correlations estimated in our study suggest that sperm chromatin fragmentation is linked to protamine deficiency and support the hypothesis that the lack of protamines contributes to sperm chromatin instability and therefore reduced fertility. Since the cause for increased DFI is largely unknown [16, 17], the reported correlations have important biological implications. One of the proposed causes of increased DFI is an inappropriate sperm chromatin structure related to protamine deficiency [18, 19]. Phenotypic correlations between sperm chromatin fragmentation and protamine deficiency were previously reported [11, 23], but here we discovered a genetic correlation between these traits.

First, we discuss the significant QTL on the X chromosome, because they stand out in these GWAS. Using the new reference assembly was important for the X chromosome (ARS-UCD1.2, https://www.ncbi.nlm.nih.gov/ assembly/GCA_002263795.2) [42] because it has recently been used to determine the boundaries of the bovine pseudo-autosomal region boundary (PAB) [45]. The known boundaries of the PAB allowed us to separate the X variants into two categories (pseudoautosomal or hemizygous) for a more accurate imputation of genotypes. No QTL mapped to the X pseudoautosomal region.

The X chromosome is conserved among mammals [57]. A recent comparison between mouse and human X chromosomes concluded that most of the genes that are not shared between species are highly expressed in testis [58]. More specifically, these species-divergent genes are expressed in the germ line of testis. Hence, Mueller et al. [58] suggested that the X chromosome is an interesting part of the genome to look for genes associated with spermatogenesis and male fertility. Our results certainly support their hypothesis, since most of the significant SNPs mapped to the X chromosome. Previous GWAS in dairy cattle also suggested that the X chromosome is important for both semen-quality traits (such as sperm concentration and motility) and bull conception rates [59, 60]. Together, these GWAS suggest that the X chromosome harbours genes that are relevant for both *Bos indicus* and *Bos taurus* bulls. However, the causative mutations may not be the same in all breeds. It is important to mention the breed-specific QTL in this discussion. The specialization of the X chromosome may continue after species divergence. An example is the QTL for MA, which appears to be unique to *Bos indicus* cattle and might be underpinned by a mutation that is absent in *Bos taurus*. The X-linked causative mutations or candidate genes might be breed-specific and difficult to pinpoint in the reported QTL, some of which span a few Mb.

Previous studies on mice identified a higher proportion of X-linked genes that are specifically expressed in spermatogonia [61]. Two current theories attempt to explain this accumulation of X-linked genes related to male germ-cell development: sex-chromosome meiotic drive and sexual antagonism. The first theory implies that some genes associated to bull fertility may also be associated with a preferential transmission of the X chromosome to gametes (instead of chromosome Y). The second theory suggests that the significant variants reported here could follow a recessive mode of inheritance, which might benefit bull fertility and not affect female fertility when they exist in the heterozygous state. Thus, according to this theory, the variants that benefit bull fertility would be deleterious to female fertility only when they exist in the homozygous state, and would be “forced” to remain at a non-deleterious frequency (and be mostly heterozygous in females). All of the peak SNPs, which were identified as the most significant markers for each QTL, could follow this “recessive mode” hypothesis because their MAF were relatively low (higher than 0.05 and lower than 0.24). At these frequencies, the alleles would likely be heterozygous in the females of these breeds.

Previous work and the current GWAS suggest that SNP *rs452801659* on the X chromosome could be an important mutation for scrotal circumference and protamine deficiency, especially in Tropical Composites [53]. However, it is worth noting that, initially, we investigated this SNP because it was predicted to be a missense variant in the *TEX11* gene (testis-specific gene), according to the previous reference genome (UMD3 assembly). In the current assembly (ARS-UCD1.2), this SNP is now an intergenic variant, outside of *TEX11* and may affect other nearby genes.

Considering the QTL that were identified for both breeds, there are two broad regions on chromosome X that harbour SNPs associated with one or another type of sperm morphological abnormality. These abnormalities are genetically and phenotypically correlated traits that may arise from the same problem: defective spermatogenesis [19, 32]. It is logical that the same gene(s) affect spermatogenesis in both breeds, thus we can prioritize as candidate genes those mapped to these consensus regions in future work.

On BTA2, it is interesting to note that the QTL peak for inhibin level is 54 kb away from the *INHA* gene that encodes inhibin alpha. Two sub-units, alpha and beta, form the inhibin hormone, which is encoded by four genes: *INHA* (chromosome 2), *INHBB* (chromosome 2), *INHBC* (chromosome 5), *INHBE* (chromosome 5), *INHBA* (chromosome 4). In short, significant and suggestive SNP associations could be pointing to genes involved in inhibin level and explain the variance in inhibin hormone level.

On BTA5, the genes *HELB* and *HMGA2* are known candidate genes in Tropical Composites. A missense mutation in *HELB* (*Bos taurus* autosome BTA5: 47,481,804–47,519,482) was associated with SC18 and SC24 in a mixed-breed analysis of bulls that are a sub-set of the same population explored here [62]. This genomic region was reported to affect climate-adaptation traits in tropical cattle and was related to a signature of selection [63, 64]. However, when the *HELB* mutation was fitted as a fixed effect, the SNPs in the same region continued to be significant [62], which suggests that the causative mutation for this QTL might be different and might affect other gene(s). The expression of *HMGA2* distinguishes between different types of post-pubertal testicular germ cell tumours [65], thus, indicating that *HMGA2* has a role in germ cell proliferation, which could affect testicular development in bulls. *HMGA2* is located within the QTL described for SC24 in our GWAS (gene start 47,819,505 and end 47,966,760 bp). Future work should test mutations in *HMGA2* for their associations with SC.

Brahman SNP associations on BTA11 serve as evidence to propose a QTL involved in sperm chromatin phenotypes, which might be recessive and requires further investigation. Sperm chromatin phenotypes are not routinely measured and the sample size available for this GWAS was small (~ 500 bulls), which limited the power of our analyses. Still, it is the first time that genetic parameters are estimated and that QTL are proposed for sperm DNA fragmentation in tropical bulls.

Visual scores and assessment of semen quality, such as motility and density, are perhaps more prone to human errors because these measures are more subjective than all the other measured phenotypes, and this could explain the lower heritability observed for these phenotypes and the extremely high correlations between all visual scores. In future studies, ideally, progressive motility should be measured with a purposed-designed software such as computer-assisted sperm analyses [66]. Future GWAS should look into this option for a more objective measurement of these important semen phenotypes.

## Conclusions

Our investigation of 25 phenotypes in two bovine breeds suggests that X-linked genes are relevant to bull fertility. The X chromosome likely evolved from an autosome and became specialized by accumulating genes and alleles that are linked to spermatogenesis. The results from this study, and previous work, support the idea that X-linked genes have orthologues across mammalian species that affect spermatogenesis and, therefore, male fertility. Furthermore, specialization of the X chromosome may have continued after divergence of *Bos* species, leading to breed-specific QTL. Low-frequency alleles beneficial to the heterozygous sex (XY) could be a consequence of their deleterious impact on female fertility (when inherited in the homozygous state). The causative mutations that underlie the identified QTL remain unresolved and the power of the current GWAS is limited for some traits (smaller numbers of animals). Nonetheless, the traits studied here (including sperm chromatin phenotypes) have a heritable component and could be targeted in selective breeding. Selective breeding for bull fertility could benefit from genomic models that include the X chromosome QTL.

## Supplementary information

**Additional file 1: Table S1.** Statistical power to detect the genetic variance of bull fertility phenotypes. Statistical power analyses for detecting the genetic variance with the genomic relationship matrices models used are provided for each measured phenotype, within each breed.

**Additional file 2.** Additional methods and results. This additional file includes details on the SPDA and SCSA methods, on the studied populations and their structure, and results for the GWAS performed. For further analyses on the structure of the Brahman population, we refer to the principal component analyses previously published [48]. The variance component model that underpins the software used accounts for sample structure in genome-wide association studies [67].

**Additional file 3: Table S2.** Estimates for SNP associations in Brahman bulls. Link to download all GWAS results (Brahman) https://doi.org/10.25919/5e670b1621b21.

**Additional file 4: Table S3.** Estimates for SNP associations in Tropical Composite bulls. Link to download all GWAS results (Tropical Composites) https://doi.org/10.25919/5e670b1621b21.

**Additional file 5: Figure S1.** Genetic correlations for 25 bull-fertility phenotypes in Brahman (above diagonal) and Tropical Composites (below diagonal). Genetic correlations were estimated using genomics and pairwise analyses for all traits, within breed. **Figure S2.** Manhattan plot for the analyses in each of the two breeds: SNP associations for the percentage of normal sperm (PNS). This figure provides a visual for the results of the genome-wide association studies in each breed, for PNS. **Figure S3.** Manhattan plot for the analyses in each of the two breeds: SNP associations for the percentage of sperm with head abnormalities (HA). This figure provides a visual for the results of the genome-wide association studies in each breed, for HA. **Figure S4.** Manhattan plot for the analyses in each of the two breeds: SNP associations for the percentage of sperm with mid-piece abnormalities (MA). This figure provides a visual for the results of the genome-wide association studies in each breed, for MA. **Figure S5.** Manhattan plot for the analyses in each of the two breeds: SNP associations for inhibin hormone levels (INH). This figure provides a visual for the results of the genome-wide association studies in each breed, for INH. **Figure S6.** Manhattan plot for the analyses in each of the two breeds: SNP associations for the DNA fragmentation index (DFI3). This figure provides a visual for the results of the genome-wide association studies in each breed, for DFI3. **Figure S7.** Manhattan plot for the analyses in each of the two breeds: SNP associations for the DNA fragmentation index measured with an alternative cytometry method (DFI4, see Methods for phenotype details). This figure provides a visual for the results of the genome-wide association studies in each breed, for DFI4. **Figure S8.** Manhattan plot for the analyses in each of the two breeds: SNP associations for the percentage of sperm with proximal droplets (PD). This figure provides a visual for the results of the genome-wide association studies in each breed, for PD. **Figure S9.** Manhattan plot for the analyses in each of the two breeds: SNP associations for the percentage of sperm with total droplets (TD), which seem specific to Tropical Composites. This figure provides a visual for the results of the genome-wide association studies in each breed, for TD. **Figure S10.** Manhattan plot for the analyses in each of the two breeds: SNP associations for scrotal circumference measured at about 24 months of age (SC24). This figure provides a visual for the results of the genome-wide association studies in each breed, for SC24. **Figure S11.** Manhattan plot for the additive (top) model and the recessive (bottom) model: SNP associations on chromosome 11 for DNA fragmentation index (DFI3) in Brahman. This figure provides a visual for the results in chromosome 11 of the SNP associations in Brahman for DFI3 using alternative models to compare the additive model with a recessive model.

## Data Availability

All GWAS data generated during this study are included in this published article and its additional files. The datasets analysed are available from the corresponding author on reasonable request.

## References

[1] Evenson DP (1999). Loss of livestock breeding efficiency due to uncompensable sperm nuclear defects. Reprod Fertil Dev.

[2] Evenson DP (2013). Sperm Chromatin Structure Assay (SCSA(R)). Methods Mol Biol.

[3] Holroyd RG, Doogan W, De Faveri J, Fordyce G, McGowan MR, Bertram JD, Vankan DM (2002). Bull selection and use in northern Australia. 4. Calf output and predictors of fertility of bulls in multiple-sire herds. Anim Reprod Sci..

[4] Fortes MR, Reverter A, Hawken RJ, Bolormaa S, Lehnert SA (2012). Candidate genes associated with testicular development, sperm quality, and hormone levels of inhibin, luteinizing hormone, and insulin-like growth factor 1 in Brahman bulls. Biol Reprod.

[5] Fortes MRS, Reverter A, Kelly M, McCulloch R, Lehnert SA (2013). Genome-wide association study for inhibin, luteinizing hormone, insulin-like growth factor 1, testicular size and semen traits in bovine species. Andrology..

[6] Fordyce G, Entwistle K, Norman S, Perry V, Gardiner B, Fordyce P (2006). Standardising bull breeding soundness evaluations and reporting in Australia. Theriogenology.

[7] Holroyd RG, Bertram JD, Doogan VJ, Fordyce G, Petherick JC, Turner LB. Breeding soundness of sale bulls after relocation. In: Bullpower: Delivery of adequate normal sperm to the site of fertilisation. Sydney: Meat and Livestock Australia (MLA); Project Report NAP3. 2004;117:11–22.

[8] Phillips DJ (2005). Activins, inhibins and follistatins in the large domestic species. Domest Anim Endocrinol.

[9] Lunstra DD, Cundiff LV (2003). Growth and pubertal development in Brahman-, Boran-, Tuli-, Belgian blue-, Hereford- and Angus-sired F1 bulls. J Anim Sci.

[10] Brito LF, Silva AE, Unanian MM, Dode MA, Barbosa RT, Kastelic JP (2004). Sexual development in early- and late-maturing *Bos indicus* and *Bos indicus x Bos taurus* crossbred bulls in Brazil. Theriogenology.

[11] Fortes MRS, Satake N, Corbet DH, Corbet NJ, Burns BM, Moore SS (2014). Sperm protamine deficiency correlates with sperm DNA damage in *Bos indicus* bulls. Andrology..

[12] Evenson D, Jost L (2000). Sperm chromatin structure assay is useful for fertility assessment. Methods Cell Sci.

[13] Boe-Hansen GB, Christensen P, Vibjerg D, Nielsen MBF, Hedeboe AM (2008). Sperm chromatin structure integrity in liquid stored boar semen and its relationships with field fertility. Theriogenology.

[14] Waterhouse KE, Haugan T, Kommisrud E, Tverdal A, Flatberg G, Farstad W (2006). Sperm DNA damage is related to field fertility of semen from young Norwegian Red bulls. Reprod Fertil Dev.

[15] Ahmadi A, Ng SC (1999). Fertilizing ability of DNA-damaged spermatozoa. J Exp Zool.

[16] Bungum M, Bungum L, Giwercman A (2011). Sperm chromatin structure assay (SCSA): a tool in diagnosis and treatment of infertility. Asian J Androl..

[17] Giwercman A, Spano M, Bungum M (2011). Sperm DNA damage: causes and guidelines for current clinical practice. Biennial Rev Infertil..

[18] Gonzalez-Marin C, Gosalvez J, Roy R (2012). Types, causes, detection and repair of DNA fragmentation in animal and human sperm cells. Int J Mol Sci.

[19] D’Occhio MJ, Hengstberger KJ, Johnston SD (2007). Biology of sperm chromatin structure and relationship to male fertility and embryonic survival. Anim Reprod Sci..

[20] Krawetz SA, Dixon GH (1988). Sequence similarities of the protamine genes—implications for regulation and evolution. J Mol Evol.

[21] Toshimori K (2003). Biology of spermatozoa maturation: an overview with an introduction to this issue. Microsc Res Techn..

[22] Balhorn R (2007). The protamine family of sperm nuclear proteins. Genome Biol.

[23] Simon L, Castillo J, Oliva R, Lewis SEM (2011). Relationships between human sperm protamines, DNA damage and assisted reproduction outcomes. Reprod Biomed Online..

[24] Evenson DP, Larson KL, Jost LK (2002). Sperm chromatin structure assay: its clinical use for detecting sperm DNA fragmentation in male infertility and comparisons with other techniques. J Androl.

[25] Miller D, Brinkworth M, Iles D (2010). Paternal DNA packaging in spermatozoa: more than the sum of its parts? DNA, histones, protamines and epigenetics. Reproduction.

[26] Hammoud SS, Nix DA, Zhang HY, Purwar J, Carrell DT, Cairns BR (2009). Distinctive chromatin in human sperm packages genes for embryo development. Nature.

[27] Puri D, Dhawan J, Mishra RK (2010). The paternal hidden agenda Epigenetic inheritance through sperm chromatin. Epigenetics..

[28] Amann RP, Seidel GE, Mortimer RG (2000). Fertilizing potential in vitro of semen from young beef bulls containing a high or low percentage of sperm with a proximal droplet. Theriogenology.

[29] Fortes MRS, Holroyd RG, Reverter A, Venus BK, Satake N, Boe-Hansen GB (2012). The integrity of sperm chromatin in young tropical composite bulls. Theriogenology.

[30] Sabeti P, Pourmasumi S, Rahiminia T, Akyash F, Talebi AR (2016). Etiologies of sperm oxidative stress. Int J Reprod Biomed (Yazd).

[31] Fischer MA, Willis J, Zini A (2003). Human sperm DNA integrity: correlation with sperm cytoplasmic droplets. Urology..

[32] Boe-Hansen GB, Fortes MRS, Satake N (2018). Morphological defects, sperm DNA integrity, and protamination of bovine spermatozoa. Andrology..

[33] van den Berg I, Boichard D, Lund MS (2016). Comparing power and precision of within-breed and multibreed genome-wide association studies of production traits using whole-genome sequence data for 5 French and Danish dairy cattle breeds. J Dairy Sci.

[34] Burns BM, Corbet NJ, Corbet DH, Crisp JM, Venus BK, Johnston DJ (2013). Male traits and herd reproductive capability in tropical beef cattle: 1. Experimental design and animal measures. Anim Prod Sci..

[35] Porto-Neto LR, Sonstegard TS, Liu GE, Bickhart DM, Da Silva MV, Machado MA (2013). Genomic divergence of zebu and taurine cattle identified through high-density SNP genotyping. BMC Genomics..

[36] Porto-Neto LR, Lehnert SA, Fortes MRS, Kelly M, Reverter A (2013). Population stratification and breed composition of Australian tropically adapted cattle. Proc Assoc Adv Anim Breed Genet..

[37] Visscher PM, Hemani G, Vinkhuyzen AA, Chen GB, Lee SH, Wray NR (2014). Statistical power to detect genetic (co)variance of complex traits using SNP data in unrelated samples. PLoS Genet.

[38] Evenson D, Darzynkiewicz Z, Jost L, Janca F, Ballachey B (1986). Changes in accessibility of DNA to various fluorochromes during spermatogenesis. Cytometry.

[39] Tavalaee M, Kiani A, Arbabian M, Deemeh MR, Esfahani MHN (2010). Flow cytometry: a new approach for indirect assessment of sperm protamine deficiency. Int J Fertil Steril..

[40] Evenson D, Jost L. Sperm chromatin structure assay for fertility assessment. Curr Protoc Cytom. 2001. Chapter 7: Unit 7. 10.1002/0471142956.cy0713s13 18770725

[41] Bolormaa S, Pryce JE, Kemper K, Savin K, Hayes BJ, Barendse W (2013). Accuracy of prediction of genomic breeding values for residual feed intake and carcass and meat quality traits in *Bos taurus*, *Bos indicus*, and composite beef cattle. J Anim Sci.

[42] Rosen BD, Bickhart DM, Schnabel RD, Koren S, Elsik CG, Tseng E (2020). De novo assembly of the cattle reference genome with single-molecule sequencing. GigaScience..

[43] Loh PR, Danecek P, Palamara PF, Fuchsberger C, Reshef YA, Finucane HK (2016). Reference-based phasing using the haplotype reference consortium panel. Nat Genet.

[44] Das S, Forer L, Schonherr S, Sidore C, Locke AE, Kwong A (2016). Next-generation genotype imputation service and methods. Nat Genet.

[45] Johnson T, Keehan M, Harland C, Lopdell T, Spelman RJ, Davis SR (2019). Short communication: identification of the pseudoautosomal region in the Hereford bovine reference genome assembly ARS-UCD1.2. J Dairy Sci..

[46] VanRaden PM (2008). Efficient methods to compute genomic predictions. J Dairy Sci.

[47] Taylor JF (2014). Implementation and accuracy of genomic selection. Aquaculture.

[48] Fortes MRS, Bolormaa S, Porto-Neto LR, Holroyd RG, Reverter A (2011). Principal component analysis in a population of Brahman bulls genotyped with 50K SNP chip revealed a genetic structure. Proc Assoc Adv Anim Breed Genet..

[49] Corbet NJ, Burns BM, Corbet DH, Crisp JM, Johnston DJ, McGowan MR (2011). Bull traits measured early in life as indicators of herd fertility. Proc Assoc Adv Anim Breed Genet..

[50] Corbet NJ, Burns BM, Corbet DH, Johnston DJ, Crisp JM, McGowan MR (2009). Genetic variation in growth, hormonal and seminal traits of young tropically adapted bulls. Proc Assoc Adv Anim Breed Genet..

[51] Corbet NJ, Burns BM, Johnston DJ, Wolcott ML, Corbet DH, Venus BK (2013). Male traits and herd reproductive capability in tropical beef cattle. 2. Genetic parameters of bull traits. Anim Prod Sci..

[52] Perez-Enciso M, Misztal I (2011). Qxpak.5: old mixed model solutions for new genomics problems. BMC Bioinformatics.

[53] de Camargo GMF, Porto-Neto LR, Kelly MJ, Bunch RJ, McWilliam SM, Tonhati H (2015). Non-synonymous mutations mapped to chromosome X associated with andrological and growth traits in beef cattle. BMC Genomics..

[54] Storgaard L, Bonde JP, Ernst E, Andersen CY, Spano M, Christensen K (2006). Genetic and environmental correlates of semen quality: a twin study. Epidemiol..

[55] Karoui S, Diaz C, Gonzalez-Marin C, Amenabar ME, Serrano M, Ugarte E (2012). Is sperm DNA fragmentation a good marker for field AI bull fertility?. J Anim Sci.

[56] Byrne TJ, Santos BFS, Amer PR, Martin-Collado D, Pryce JE, Axford M (2016). New breeding objectives and selection indices for the Australian dairy industry. J Dairy Sci.

[57] Delgado CLR, Waters PD, Gilbert C, Robinson TJ, Graves JAM (2009). Physical mapping of the elephant X chromosome: conservation of gene order over 105 million years. Chromosome Res.

[58] Mueller JL, Skaletsky H, Brown LG, Zaghlul S, Rock S, Graves T (2013). Independent specialization of the human and mouse X chromosomes for the male germ line. Nat Genet.

[59] Blaschek M, Kaya A, Zwald N, Memili E, Kirkpatrick BW (2011). A whole-genome association analysis of noncompensatory fertility in Holstein bulls. J Dairy Sci.

[60] Suchocki T, Szyda J (2015). Genome-wide association study for semen production traits in Holstein-Friesian bulls. J Dairy Sci.

[61] Wang PJ, Pan J (2007). The role of spermatogonially expressed germ cell-specific genes in mammalian meiosis. Chromosome Res.

[62] Fortes MRS, Almughlliq FB, Nguyen LT, Porto-Neto LR, Lehnert SA. Non-synonymous polymorphism in *HELB* is associated with male and female reproductive traits in cattle. In Proceedings of the 21st conference of the association for the advancement of animal breeding and genetics: 28–30 September 2015; Lorne; 2015. p. 73–6.

[63] Porto-Neto LR, Reverter A, Prayaga KC, Chan EK, Johnston DJ, Hawken RJ (2014). The genetic architecture of climatic adaptation of tropical cattle. PLoS One.

[64] Naval-Sánchez M, Porto-Neto LR, Cardoso DF, Hayes BJ, Daetwyler HD, Kijas J (2020). Selection signatures in tropical cattle are enriched for promoter and coding regions and reveal missense mutations in the damage response gene *HELB*. Genet Sel Evol..

[65] Kloth L, Gottlieb A, Helmke B, Wosniok W, Loning T, Burchardt K (2015). *HMGA2* expression distinguishes between different types of postpubertal testicular germ cell tumour. J Pathol Clin Res..

[66] Amann RP, Waberski D (2014). Computer-assisted sperm analysis (CASA): capabilities and potential developments. Theriogenology.

[67] Kang HM, Sul JH, Service SK, Zaitlen NA, Kong S, Freimer NB (2010). Variance component model to account for sample structure in genome-wide association studies. Nat Genet..

