# Supplementary material for: X chromosome variants are associated with male fertility traits in two bovine populations
Source: Genet Sel Evol. 2020 Aug 12;52:46. doi: 10.1186/s12711-020-00563-5 (PMC7425018; doi:10.1186/s12711-020-00563-5)
Supplement: Supplementary file 1 — Additional file 1: Table S1. Statistical power to detect the genetic variance of bull fertility phenotypes. Statistical power analyses for detecting the genetic variance with the genomic relationship matrices models used are provided for each measured phenotype, within each breed. [file 12711_2020_563_MOESM1_ESM.docx]

**Additional file 1**

Table S1 Statistical power to detect the genetic variance of bull fertility phenotypes

| Phenotypes | | Brief description | Brahman | | | | | Tropical Composites | | | | |
| --- | --- | --- | --- | --- | --- | --- | --- | --- | --- | --- | --- | --- |
|  |  |  | N | *h^2^* | SE | NCP | Power | N | *h^2^* | SE | NCP | Power |
| Group 1 | PNS | Percentage of normal sperm, % | 1 023 | 0.35 | 0.07 | 152.43 | 1.00 | 1 648 | 0.29 | 0.05 | 248.82 | 1.00 |
|  | PD | Sperm with proximal droplets, % | 1 023 | 0.35 | 0.07 | 152.43 | 1.00 | 1 648 | 0.21 | 0.04 | 130.47 | 1.00 |
|  | DD | Sperm with distal droplets, % | 1 023 | 0.10 | 0.05 | 12.44 | 0.59 | 1 648 | 0.13 | 0.04 | 50.00 | 0.99 |
|  | TD | Sperm with droplets (total), % | 1 023 | 0.33 | 0.07 | 135.51 | 1.00 | 1 648 | 0.18 | 0.04 | 95.86 | 1.00 |
|  | HA | Sperm with head abnormalities, % | 1 023 | 0.27 | 0.06 | 90.71 | 1.00 | 1 648 | 0.30 | 0.05 | 266.28 | 1.00 |
|  | MA | Sperm with midpiece abnormalities, % | 1 023 | 0.07 | 0.05 | 6.10 | 0.21 | 1 648 | 0.21 | 0.05 | 130.47 | 1.00 |
|  | TA | Sperm with abnormal tail, % | 1 023 | 0.00 | 0.03 | 0.00 | 0.001 | 1 648 | 0.00 | 0.02 | 0.00 | 0.001 |
|  | COL | Color, visual score 1–5 | 1 099 | 0.09 | 0.05 | 11.63 | 0.55 | 1 719 | 0.05 | 0.03 | 8.05 | 0.33 |
|  | MOT | Progressive sperm motility, % | 1 099 | 0.00 | 0.04 | 0.00 | 0.001 | 1 719 | 0.13 | 0.04 | 54.40 | 1.00 |
|  | MAS | Mass activity, visual score 1–5 | 1 099 | 0.09 | 0.05 | 11.63 | 0.55 | 1 719 | 0.12 | 0.04 | 46.35 | 0.99 |
|  | CON | Sperm concentration, x10^6^/ml | 592 | 0.03 | 0.06 | 0.38 | 0.004 | 538 | 0.04 | 0.07 | 0.50 | 0.005 |
|  | DEN | Density, visual score 1–5 | 1 098 | 0.13 | 0.06 | 24.23 | 0.95 | 1 716 | 0.08 | 0.03 | 20.53 | 0.89 |
| Group 2 | Inhibin | Blood levels of inhibin at four months, (ng/ml) | 806 | 0.63 | 0.08 | 306.57 | 1.00 | 1 329 | 0.56 | 0.06 | 603.39 | 1.00 |
|  | SC12 | SC at 12 months, cm | 1 098 | 0.57 | 0.06 | 465.73 | 1.00 | 1 717 | 0.65 | 0.04 | 1356.88 | 1.00 |
|  | SC18 | SC at 18 months, cm | 1 098 | 0.61 | 0.06 | 533.39 | 1.00 | 1 719 | 0.67 | 0.04 | 1445.02 | 1.00 |
|  | SC24 | SC at 24 months, cm | 1 098 | 0.63 | 0.06 | 568.94 | 1.00 | 1 719 | 0.70 | 0.04 | 1577.33 | 1.00 |
| Group 3 | PIC3 | Sperm with intact chromatin in FL3, % | 585 | 0.12 | 0.08 | 5.86 | 0.19 | 511 | 0.16 | 0.09 | 7.28 | 0.28 |
|  | DFI3 | DNA fragmentation index in FL3, % | 585 | 0.10 | 0.07 | 4.00 | 0.10 | 511 | 0.21 | 0.10 | 12.54 | 0.60 |
|  | HDS3 | High DNA stainability in FL3, % | 585 | 0.05 | 0.07 | 1.02 | 0.01 | 511 | 0.20 | 0.11 | 11.38 | 0.53 |
|  | PIC4 | Sperm with intact chromatin in FL4, % | 585 | 0.17 | 0.08 | 11.76 | 0.56 | 511 | 0.17 | 0.09 | 8.22 | 0.34 |
|  | DFI4 | DNA fragmentation index in FL4, % | 585 | 0.15 | 0.09 | 9.16 | 0.40 | 511 | 0.15 | 0.09 | 6.40 | 0.22 |
|  | HDS4 | High DNA stainability in FL4, % | 585 | 0.04 | 0.07 | 0.65 | 0.01 | 511 | 0.20 | 0.11 | 11.38 | 0.53 |
| Group 4 | LCB | Low CMA3 binding (intact protamination), % | 592 | 0.22 | 0.09 | 20.17 | 0.89 | 538 | 0.13 | 0.08 | 5.33 | 0.16 |
|  | MCB | Medium CMA3 binding (medium protamination), % | 592 | 0.17 | 0.08 | 12.04 | 0.57 | 538 | 0.13 | 0.09 | 5.33 | 0.16 |
|  | HCB | High CMA3 binding (protamine deficiency), % | 592 | 0.01 | 0.06 | 0.04 | 0.001 | 538 | 0.07 | 0.08 | 5.33 | 0.02 |

N: number of animals in the analysis; *h^2^*: phenotype SNP-heritability; SE: standard error of the SNP-heritability; NCP: non-centrality parameter of the chi-squared test statistic, which is equal to *h^4^/(SE)^2^*; Power: the probability of detecting *h^2^* > 0 using 0.001 as the specified type I error rate and the SNP-heritability estimated for these two populations of cattle.
