# Supplementary material for: X chromosome variants are associated with male fertility traits in two bovine populations
Source: Genet Sel Evol. 2020 Aug 12;52:46. doi: 10.1186/s12711-020-00563-5 (PMC7425018; doi:10.1186/s12711-020-00563-5)
Supplement: Supplementary file 2 — Additional file 2. Additional methods and results. This additional file includes details on the SPDA and SCSA methods, on the studied populations and their structure, and results for the GWAS performed. For further analyses on the structure of the Brahman population, we refer to the principal component analyses previously published [48]. The variance component model that underpins the software used accounts for sample structure in genome-wide association studies [67]. [file 12711_2020_563_MOESM2_ESM.docx]

Additional file 2

Additional Methods

Sperm Protamine Deficiency Assay (SPDA)

The percentages of sperm cells with high-, medium-, and low-protamine content (HPC, MPC, and LPC) were estimated with SPDA, and details about this flow cytometric methodology were provided in previous studies [11, 32]. Briefly, sperm protamine was assessed using CMA3, which is a fluorochrome that competes for protamine-binding sites at the minor groove of a DNA strand [38, 39]. Samples were thawed (37°C for three minutes) and sperm concentration was determined using the Improved Neubauer chamber. Then, samples were diluted to approximately 50$\times$10^6^ sperm/mL in Dulbecco’s phosphate-buffered saline (DPBS, Ca^++^, and Mg^++^ free), and subsequently washed by centrifugation (500 g for five minutes as for all centrifugation washes). The pellet was resuspended in DPBS, and then split into two aliquots: (1) without treatment and (2) a positive control treated with 5 mM dithiothreitol (DTT) and incubated for 15 minutes at 37°C. Then, samples were washed twice by centrifugation, resuspended in DPBS, washed again, resuspended in 0.25 mg/mL CMA3 in McIlvaine’s buffer (17 mM citric acid, 164 mM Na_2_HPO_4_, and 10 mM MgCl_2_6H_2_O; pH 7.0), and finally incubated at room temperature for one hour in complete darkness. Again, the samples were washed in DPBS three times by centrifugation and the concentration was adjusted to 5–10$\times$10^6^ sperm/mL for flow cytometric analysis on the Beckman Coulter Gallios flow cytometer. Excitation solid-state lasers, violet lasers (405 nm), and blue lasers (488 nm) were used, and fluorescence was detected on FL9 (450BP50 filter), FL10 (550BP40 filter), and FL2 (575BP30 filter), respectively, at a low-flow rate. In addition, the Kaluza software (version 1.1) was used to analyze the flow cytometric data and generate the three phenotypes for genetic analyses: HPC, MPC, and LPC.

Sperm Chromatin Structure Assay (SCSA)

Samples thawed for SPDA were used for SCSA, which measured three related phenotypes: percentage of PIC, DFI, and percentage of sperm with abnormal HDS. This process was conducted according to the protocol described by Evenson and Jost [40] using the metachromatic properties of acridine orange to assess chromatin stability. This compound fluoresces green when combined with double-stranded (intact) DNA, and fluoresces red when combined with single-stranded (fragmented) DNA;, the flow cytometric analyses for SCSA used the same equipment, as described by our group previously [11].

When using the blue excitation laser (488 nm) to excite the fluorophore, acridine orange and fluorescence were detected on FL1 (525BP40 filter), FL3 (620BP30 filter), and FL4 (675BP20 filter) at a low-flow rate. After every six test samples, a reference sample was thawed and analyzed to ensure stability of the instrument. The Kaluza software (version 1.1) was used to analyze the flow cytometric data and generate the three phenotypes for genetic analyses: PIC, DFI and HDS. Two DFI values were determined using the FL3 fluorescence (DFI3) and FL4 fluorescence (DFI4) for detecting sperm with DNA damage. Likewise, HDS was determined using the two filters FL3 (HDS3) and FL4 (HDS4). The use of the Gallios cytometer and software, which adds an FL4 reading to the standard SCSA, was previously developed [29].

*Genome-wide Association Studies (GWAS)*

To perform GWAS, we built a GRM for each breed using all imputed SNPs that passed quality control and had a MAF higher than 0.05 (within breed). Each GRM was built using the first method proposed by VanRaden [46], which is a GBLUP method. We used all the default parameters in the SNP & Variation Suite (SVS) software (release 8.3.0, Golden Helix), including the overall normalization method, as described by Taylor [47]. We also used a correction for sex, by informing the program that all animals were bulls and selecting for full dosage compensationon the X chromosome. The estimated relationships for both breeds (the GRM off-diagonal elements) had a variance of ~0.002. These relationships estimated from genotypes conform with the expectations of having measured the progeny of 55 Brahman sires and 56 Tropical Composite sires. We used these precomputed GRM (including the X variants) to fit the random polygenic effect in all our models.

GWAS were carried out using an additive mixed model to compute single-trait-single-SNP associations. All 722,208 available SNPs were tested individually for each phenotype, in each breed. GWAS carried out within breed allowed the detection of breed-specific QTL, which were expected for these populations [4, 5] as shown in the results discussed above. The GWAS models used the precomputed GRM, contemporary groups as fixed effects and age as a covariant. The effects of contemporary groups and age for the measured traits were examined in previous studies [4, 5, 49, 50, 51]. Contemporary groups were defined as cohorts of bulls that were born in the same year and raised together in the same location. SNP additive effects for each trait were calculated by fitting these mixed models in the SVS software (release 8.3.0, Golden Helix). This software uses the model proposed by Kang et al. [67], which included a genomic best linear unbiased prediction (gBLUP) and fits the GRM, as follows:

$\mathbf{y=X}\beta\boldsymbol{+sa+u+\varepsilon}$,

where, $\boldsymbol{X}$ is the incidence matrix of fixed effects in $\beta$ (including cohorts). The bull age was fitted as a covariant (i.e. the age in days when the trait was measured). In addition, $\mathbf{s}$ is the vector with the genotypes codified as 0, 1, or 2 according to the number of allele *B* copies, $\mathbf{a}$ is the vector containing the SNP effects, $\mathbf{u}$ is the vector of polygenic random effects, and $\boldsymbol{\varepsilon}$ is the vector of residuals. Both $\mathbf{u}$ and $\boldsymbol{\varepsilon}$ follow normal distribution with ($\mathbf{u}\sim N(\boldsymbol{0},\mathbf{G}\sigma_{a}^{2}$*)*) and ($\boldsymbol{\varepsilon}\sim N(\mathbf{0},\mathbf{I}\sigma_{e}^{2}$*)*), respectively, where $\mathbf{G}$ is the GRM for all individual $\sigma_{a}^{2}$ is the additive genetic variance, $\mathbf{I}$ is an identity matrix, and $\sigma_{e}^{2}$ is the residual variance. These analyses were performed with the Golden Helix software, which allowed to fit both additive and non-additive models. For the additive model, the software recodes the major homozygous genotype to 0, the heterozygous to 1, and the minor homozygous to 2. For the dominant model, the genotypes are recoded as follows: major homozygous genotype to 0 and both heterozygous and minor homozygous to 1. For the recessive model, the genotypes are recoded as: major homozygous and heterozygous recoded to 0 and minor homozygous recoded to 1. First, we fit the additive model for all GWAS. Next, when autosomal QTL were identified, both dominant and recessive models were tested. Non-additive models were used to perform GWAS for inhibin level and DFI3 in Brahman, since QTL were identified on chromosomes 2 and 11 for these phenotypes. In Tropical Composites, non-additive models tested SNP associations for SC24 on chromosome 5. In all models, we used the same precomputed GRM.

The precomputed GRM was also used to estimate genetic correlations and heritability for the studied phenotypes, within breed. Genetic correlations were estimated by fitting bivariate genomic mixed models using the Qxpak5 software [52]. Fixed effects and covariants were the same as mentioned above and the random polygenic effect was estimated from the precomputed GRM. The standard errors for heritability were estimated twice: first, with the Golden Helix software simultaneously with the estimates for heritability (same model), and second, with the power calculations described above, derived with Visscher’s method [37].

To report on significant SNPs, MAF was considered and the results for SNPs with MAF lower than 0.05 (within breed) were not deemed significant regardless of the estimated *P*-values. The significant SNP reported for each trait followed the thresholds that we established: a *P* < 10^-8^ and a MAF higher than 0.05, which is a conservative Bonferroni correction, because it considered all SNPs as independent tests (*P* = 7 x 10^-8^ equivalent to *P* = 0.05 when considering 722,208 tests).

Additional Results

***Computing the Genomic Relationship Matrix***

When computing the Genomic Relationship Matrix (GRM) for each breed, we identified population structures that were expected base on pedigree data for these animals (many halfsibs). The Brahman population was formed by 55 sire families as illustrated before [48] and the Tropical Composites were formed by 56 sire families [5]. For each breed, the GRM was constructed using all SNPs that passed the quality control (described in the main text) by applying the first method proposed by VanRaden [46] and using the overall normalization and correcting for sex [47] (see the methods section below for more details). The summary statistics of each GRM are discussed below.

In Brahman, the diagonal elements of the GRM had a mean of 1.00577 and a standard deviation of 0.04367 (minimun and maximum numbers were 0.89383 and 1.23434) with a variance of 0.00191. The off-diagonal elements of the Brahman GRM had a mean of -0.00092 and a standard deviation of 0.04876 (minimun and maximum numbers were -0.09646 and 0.59431) with a variance of 0.00238.

In Tropical Composites, the diagonal elements of the GRM had a mean of 1.0036 and a standard deviation of 0.03574 (minimun and maximum numbers were 0.91839 and 1.17518) with a variance of 0.00128. The off-diagonal elements of the GRM for Tropical Composites had a mean of -0.00058 and a standard deviation of 0.04747 (minimun and maximum numbers were -0.13056 and 0.58294) with a variance of 0.00225.

The variance of these SNP-derived relationships (off-diagonals) were used to calculate empirically the power of the GWAS. Power analyses used the methods derived by Visscher’s group, with a word of caution: their methods were optimized for unrelated human populations [37]. The results for the power analyses are in Table S1 [see Additional file 1 Table S1]. On a scale from 0 to 1, the power for all the presented GWAS ranged from 0.001 to 1. To be more cautious when reporting on power analyses, we used a stricter cut-off for type one errror as discussed in the main text. The SE for the heritability estimated with the power analyses were lower than the reported estimates, which were from the complete model for the GWAS, performed with Golden Helix. The SE from the power analyses ranged from 0.02 to 0.06, while SE reported in this paper (calculated from our models fit in the Golden Helix software) ranged from 0.02 to 0.11. Naturally, errors were larger when the sample size was smaller. Visscher and colleagues report that their power analyses could be affected by population structure if the SE of heritability estimates were lower than their error estimates, which is not the case for our populations.

***Genetic Correlations from Bivariate Analyses***

Genetic correlations across traits were estimated for Brahman and Tropical Composite bulls [See Additional file 5 Figure S1]. In absolute values, genetic correlations ranged from as low as 0 (e.g., between TA and PD in Brahman) to as high as 0.999 (e.g., between HDS4 and HDS3 in Tropical Composites). These genetic correlations are as presented in Fig. 1 in the main text; hence, including them here provides all the numerical values estimated for each correlation. It should be noted that the phenotypes from SCSA and SPDA were available only for a sub-set of the bulls since these phenotypes are not routinaly measured (n = 592 for Brahman and n = 538 for Tropical Composites). These phenotypes were described before, in a prior publication by Boe-Hansen and colleagues that focussed only on the phenotypes and phenotypic correlations [32].

***Genome-wide Association Studies for each phenotype***

Significant associations at the *P*-value equal to 1$\times$10^-8^ level (as reported in the main text) point to the QTL proposed in this study. The Manhattan plots provided in Figures S2 to S10 [see Additional file 5 Figures S2 to S10] allow a visual inspection of each QTL in a genome-wide context. We show the results from the two breeds side-by-side to support the idea that some QTL are breed-specific, while others may occur in both breeds. It is evident from these figures that the proposed QTL are supported by association peaks that are typical of robust GWAS signals.

For the SNP associations on autosomes, we also used non-additive models to verify if any recessive or dominant effects could explain the proposed QTL. In Brahman, two autosomal QTL were detected (see main text, Fig. 2): a QTL for inhibin level on chromosome 2 and a QTL for DFI3 on chromosome 11. In Tropical Composites, the only autosomal QTL was associated with SC24 on chromosome 5. The QTL for inhibin level in Brahman on chromosome 2 seemed less significant when non-additive models were used. The same was true for the SC24 QTL on chromosome 5 in Tropical composites. The only case for which the recessive model was able to capture more extreme SNP associations (lowest *P*-value = 3.38$\times$10^-30^; peak SNP was located at 11:105,086,184 bp) was for the DFI3 QTL on chromosome 11. Note that the peak SNP for DF13 is different between the additive model and the recessive model [see Additional file 5 Figure S11].
