# Supplementary material for: X chromosome variants are associated with male fertility traits in two bovine populations
Source: Genet Sel Evol. 2020 Aug 12;52:46. doi: 10.1186/s12711-020-00563-5 (PMC7425018; doi:10.1186/s12711-020-00563-5)
Supplement: Supplementary file 5 — Additional file 5: Figure S1. Genetic correlations for 25 bull-fertility phenotypes in Brahman (above diagonal) and Tropical Composites (below diagonal). Genetic correlations were estimated using genomics and pairwise analyses for all traits, within breed. Figure S2. Manhattan plot for the analyses in each of the two breeds: SNP associations for the percentage of normal sperm (PNS). This figure provides a visual for the results of the genome-wide association studies in each breed, for PNS. Figure S3. Manhattan plot for the analyses in each of the two breeds: SNP associations for the percentage of sperm with head abnormalities (HA). This figure provides a visual for the results of the genome-wide association studies in each breed, for HA. Figure S4. Manhattan plot for the analyses in each of the two breeds: SNP associations for the percentage of sperm with mid-piece abnormalities (MA). This figure provides a visual for the results of the genome-wide association studies in each breed, for MA. Figure S5. Manhattan plot for the analyses in each of the two breeds: SNP associations for inhibin hormone levels (INH). This figure provides a visual for the results of the genome-wide association studies in each breed, for INH. Figure S6. Manhattan plot for the analyses in each of the two breeds: SNP associations for the DNA fragmentation index (DFI3). This figure provides a visual for the results of the genome-wide association studies in each breed, for DFI3. Figure S7. Manhattan plot for the analyses in each of the two breeds: SNP associations for the DNA fragmentation index measured with an alternative cytometry method (DFI4, see Methods for phenotype details). This figure provides a visual for the results of the genome-wide association studies in each breed, for DFI4. Figure S8. Manhattan plot for the analyses in each of the two breeds: SNP associations for the percentage of sperm with proximal droplets (PD). This figure provides a visual for the results of the genome-wide [file 12711_2020_563_MOESM5_ESM.docx]

Additional file 5

Additional Figures S1 – S11

| Traits | PNS | PD | DD | TD | HA | MA | TA | COL | MOT | MAS | CON | DEN | Inhibin | SC12 | SC18 | SC24 | PIC3 | DFI3 | HDS3 | PIC4 | DFI4 | HDS4 | LCB | MCB | HCB |
| --- | --- | --- | --- | --- | --- | --- | --- | --- | --- | --- | --- | --- | --- | --- | --- | --- | --- | --- | --- | --- | --- | --- | --- | --- | --- |
| PNS |  | -0.696 | -0.161 | -0.734 | -0.534 | -0.468 | -0.066 | 0.272 | 0.546 | 0.465 | 0.068 | 0.292 | -0.068 | 0.085 | 0.224 | 0.131 | 0.055 | -0.211 | 0.061 | 0.185 | -0.325 | 0.220 | 0.250 | -0.251 | -0.175 |
| PD | -0.597 |  | -0.024 | 0.968 | 0.053 | -0.020 | -0.037 | -0.375 | -0.192 | -0.342 | -0.156 | -0.330 | 0.001 | -0.209 | -0.481 | -0.246 | 0.022 | 0.066 | -0.305 | -0.026 | 0.109 | -0.316 | -0.142 | 0.149 | 0.149 |
| DD | -0.153 | 0.003 |  | 0.268 | -0.054 | -0.041 | -0.012 | -0.075 | 0.053 | -0.102 | -0.081 | -0.089 | 0.016 | -0.022 | -0.009 | -0.044 | 0.081 | -0.047 | -0.075 | 0.053 | -0.006 | -0.025 | 0.045 | -0.046 | -0.016 |
| TD | -0.603 | 0.816 | 0.523 |  | 0.036 | -0.030 | -0.035 | -0.343 | -0.179 | -0.381 | -0.174 | -0.411 | 0.005 | -0.222 | -0.509 | -0.272 | 0.035 | 0.072 | -0.291 | -0.017 | 0.115 | -0.301 | -0.108 | 0.115 | 0.127 |
| HA | -0.789 | 0.300 | -0.059 | 0.062 |  | 0.032 | -0.003 | 0.044 | -0.447 | -0.286 | 0.153 | 0.024 | 0.100 | 0.138 | 0.126 | 0.099 | -0.097 | 0.250 | -0.031 | -0.249 | 0.387 | -0.029 | -0.308 | 0.306 | 0.190 |
| MA | -0.533 | 0.143 | -0.024 | 0.148 | -0.019 |  | 0.105 | -0.091 | -0.223 | -0.070 | -0.047 | -0.123 | 0.022 | -0.009 | -0.036 | -0.008 | -0.083 | 0.095 | 0.048 | -0.104 | 0.100 | -0.018 | -0.011 | 0.003 | -0.043 |
| TA | -0.023 | -0.031 | -0.033 | -0.011 | -0.007 | -0.030 |  | -0.221 | 0.012 | 0.005 | -0.001 | -0.152 | 0.007 | -0.033 | 0.105 | 0.023 | 0.000 | -0.016 | 0.011 | 0.001 | -0.014 | 0.015 | 0.005 | -0.010 | -0.011 |
| COL | 0.069 | -0.195 | -0.098 | -0.202 | 0.044 | -0.039 | 0.060 |  | 0.294 | 0.590 | 0.384 | 0.961 | 0.036 | 0.134 | 0.209 | 0.144 | -0.058 | -0.067 | 0.116 | 0.018 | -0.144 | 0.118 | 0.085 | -0.057 | -0.069 |
| MOT | 0.358 | -0.216 | 0.023 | -0.189 | -0.321 | -0.116 | -0.012 | 0.417 |  | 0.738 | 0.016 | 0.306 | -0.001 | 0.059 | 0.124 | 0.130 | 0.261 | -0.300 | -0.149 | 0.351 | -0.367 | -0.137 | 0.240 | -0.237 | -0.103 |
| MAS | 0.305 | -0.231 | -0.026 | -0.174 | -0.371 | 0.026 | 0.069 | 0.629 | 0.690 |  | 0.233 | 0.632 | 0.040 | 0.142 | 0.212 | 0.168 | 0.090 | -0.246 | 0.014 | 0.184 | -0.305 | 0.020 | 0.183 | -0.168 | -0.217 |
| CON | 0.050 | -0.123 | -0.115 | -0.172 | 0.050 | -0.009 | -0.038 | 0.270 | 0.119 | 0.259 |  | 0.424 | 0.102 | 0.184 | 0.201 | 0.172 | -0.018 | -0.040 | 0.048 | 0.017 | -0.069 | 0.045 | -0.032 | 0.055 | 0.000 |
| DEN | 0.065 | -0.134 | -0.148 | -0.194 | 0.036 | -0.040 | 0.006 | 0.894 | 0.435 | 0.663 | 0.333 |  | 0.056 | 0.137 | 0.213 | 0.156 | -0.100 | -0.036 | 0.150 | -0.028 | -0.111 | 0.152 | 0.066 | -0.038 | -0.071 |
| Inhibin | -0.202 | 0.073 | -0.144 | -0.041 | 0.149 | 0.176 | -0.126 | -0.171 | -0.032 | -0.108 | -0.011 | -0.092 |  | 0.228 | 0.231 | 0.276 | 0.014 | 0.043 | -0.048 | -0.005 | 0.053 | -0.046 | -0.031 | 0.041 | 0.026 |
| SC12 | 0.247 | -0.271 | -0.063 | -0.223 | -0.211 | -0.006 | 0.098 | 0.089 | 0.109 | 0.150 | 0.153 | 0.093 | 0.038 |  | 0.891 | 0.663 | -0.062 | 0.004 | -0.035 | 0.003 | 0.026 | -0.022 | -0.042 | 0.046 | -0.051 |
| SC18 | 0.178 | -0.292 | -0.061 | -0.242 | -0.103 | 0.004 | 0.095 | 0.160 | 0.117 | 0.164 | 0.133 | 0.151 | 0.113 | 0.885 |  | 0.846 | -0.095 | 0.003 | -0.016 | -0.017 | 0.041 | -0.010 | -0.084 | 0.092 | -0.008 |
| SC24 | 0.125 | -0.164 | -0.022 | -0.147 | -0.083 | 0.004 | 0.034 | 0.132 | 0.079 | 0.122 | 0.090 | 0.095 | 0.119 | 0.794 | 0.915 |  | 0.033 | -0.038 | -0.021 | -0.092 | -0.015 | -0.018 | -0.099 | 0.116 | 0.008 |
| PIC3 | 0.266 | -0.122 | -0.059 | -0.143 | -0.252 | -0.050 | 0.066 | 0.054 | 0.174 | 0.166 | 0.054 | 0.019 | -0.026 | -0.068 | -0.053 | -0.048 |  | -0.657 | -0.880 | 0.896 | -0.435 | -0.868 | 0.193 | -0.153 | -0.141 |
| DFI3 | -0.324 | 0.198 | 0.096 | 0.221 | 0.294 | 0.031 | 0.075 | -0.110 | -0.206 | -0.191 | -0.212 | -0.089 | 0.051 | -0.113 | -0.052 | 0.089 | -0.828 |  | 0.306 | -0.711 | 0.818 | 0.273 | -0.311 | 0.302 | 0.301 |
| HDS3 | -0.069 | -0.028 | -0.012 | -0.018 | 0.082 | 0.048 | -0.061 | 0.038 | -0.120 | -0.059 | 0.159 | 0.073 | -0.016 | 0.129 | 0.190 | 0.128 | -0.746 | 0.123 |  | -0.726 | 0.048 | 0.998 | -0.054 | 0.008 | -0.009 |
| PIC4 | 0.284 | -0.155 | -0.066 | -0.161 | -0.272 | -0.024 | 0.054 | 0.099 | 0.180 | 0.208 | 0.103 | 0.077 | -0.072 | 0.086 | 0.028 | 0.012 | 0.676 | -0.784 | -0.393 |  | -0.718 | -0.715 | 0.308 | -0.271 | -0.210 |
| DFI4 | -0.272 | 0.177 | 0.078 | 0.180 | 0.255 | 0.006 | -0.035 | -0.122 | -0.230 | -0.201 | -0.171 | -0.111 | 0.083 | -0.141 | -0.086 | -0.045 | -0.548 | 0.713 | 0.046 | -0.937 |  | 0.029 | -0.395 | 0.389 | 0.317 |
| HDS4 | -0.072 | -0.026 | -0.015 | -0.017 | 0.086 | 0.050 | -0.061 | 0.038 | -0.123 | -0.062 | 0.159 | 0.073 | -0.015 | 0.129 | 0.180 | 0.119 | -0.740 | 0.119 | 0.999 | -0.385 | 0.036 |  | -0.046 | -0.002 | -0.027 |
| LCB | 0.298 | -0.167 | -0.054 | -0.149 | 0.051 | -0.060 | 0.067 | 0.094 | 0.171 | 0.225 | 0.183 | 0.080 | -0.059 | 0.031 | 0.035 | 0.052 | 0.419 | -0.493 | -0.204 | 0.483 | -0.507 | -0.202 |  | -0.981 | -0.746 |
| MCB | -0.290 | 0.168 | 0.054 | 0.150 | 0.253 | 0.061 | -0.067 | -0.094 | -0.291 | -0.222 | -0.182 | -0.081 | 0.062 | 0.005 | -0.034 | -0.049 | -0.455 | 0.412 | 0.203 | -0.536 | 0.404 | 0.201 | -0.999 |  | 0.785 |
| HCB | -0.242 | 0.096 | 0.034 | -0.019 | 0.256 | -0.030 | -0.007 | -0.088 | -0.273 | -0.242 | -0.226 | -0.099 | 0.026 | -0.069 | -0.051 | -0.032 | -0.420 | 0.426 | 0.145 | -0.469 | 0.387 | 0.149 | -0.813 | 0.752 |  |

**Fig S1**. Genetic correlations for 25 bull-fertility phenotypes in Brahman (**above** **diagonal**) and Tropical Composites (**below diagonal**). Negative genetic correlations are presented in red, while positive ones are presented in blue. Darker shades of red or blue correspond to more extreme correlations (closer to 1 or –1), while pastel shades correspond to lower genetic correlations. The numerical values represented herein are the underpining values for Fig 1 in the main text. Abbreviations are described in the main text (see Table 1).


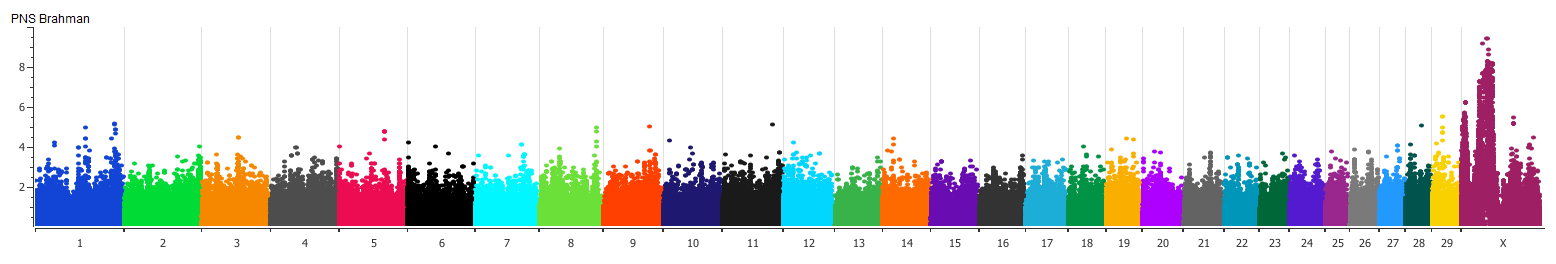


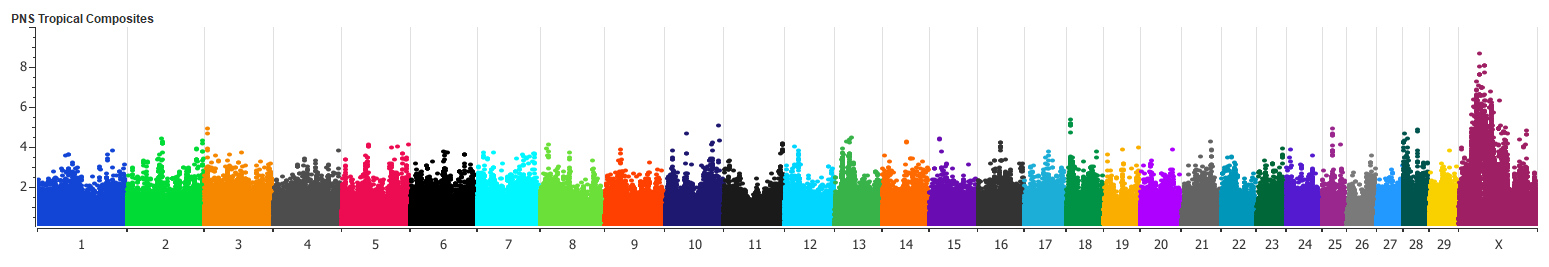


**Fig S2.** Manhattan plot for the analyses in each of the two breeds: SNP associations for the percentage of normal sperm (PNS). Despite breed differences, it is possible to observe that SNP in the X chromosome seem important for PNS in both breeds.

**
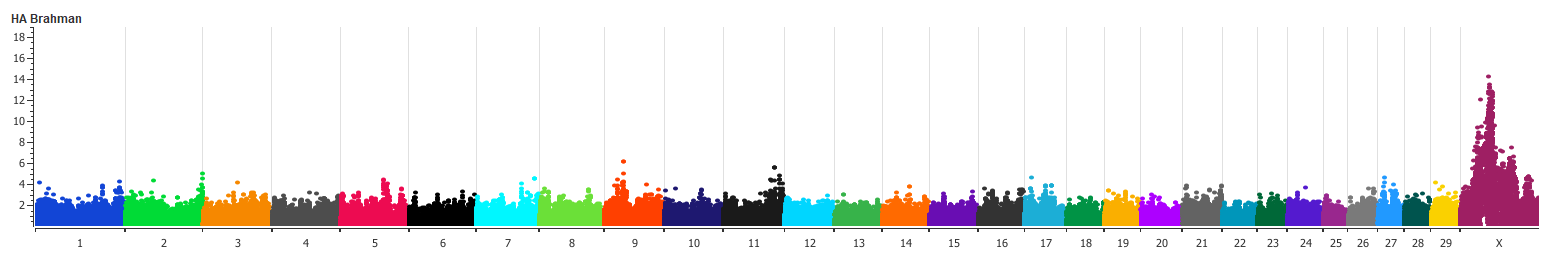

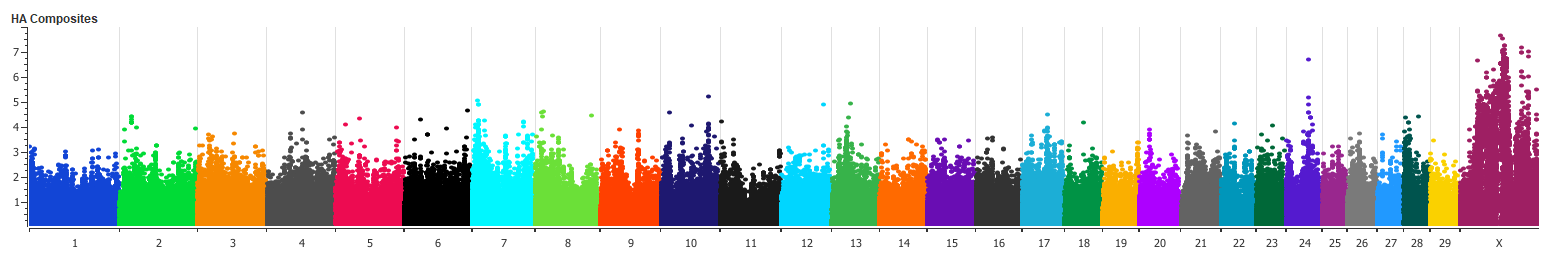
**

**Fig S3.** Manhattan plot for the analyses in each of the two breeds: SNP associations for the percentage of sperm with head abnormalities (HA). The QTL for HA was defined because of the highly significant SNP associations in that breed. Still, this QTL might be relevant to Tropical Composites as well, since SNP associations can be seen in that breed too.

**
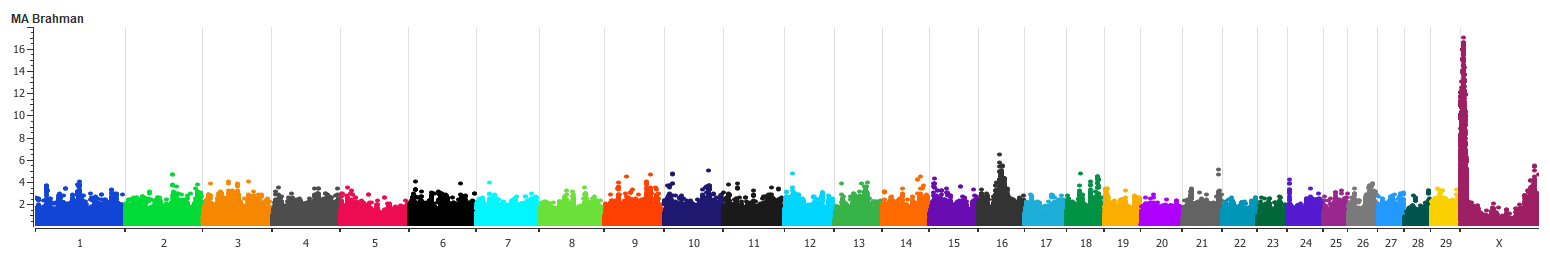
**

**
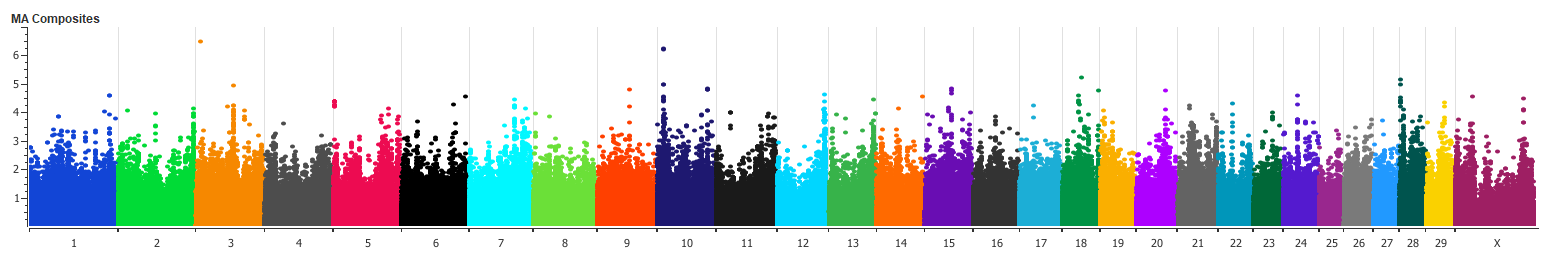
**

**Fig S4.** Manhattan plot for the analyses in each of the two breeds: SNP associations for the percentage of sperm with mid-piece abnormalities (MA). Note that the association peak in chrosome X occurred only in Brahman, with not even suggestive associations for the Tropical Composite GWAS. These results were also shown in the main text, as an example of breed specific QTL.

**
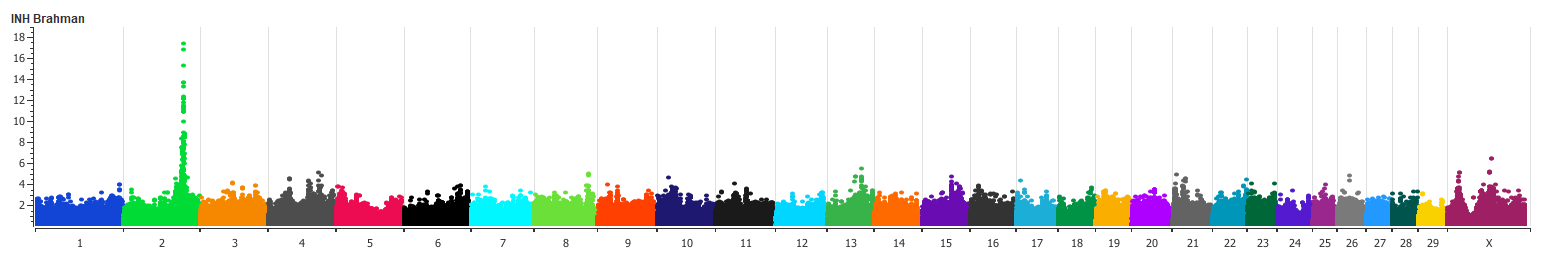

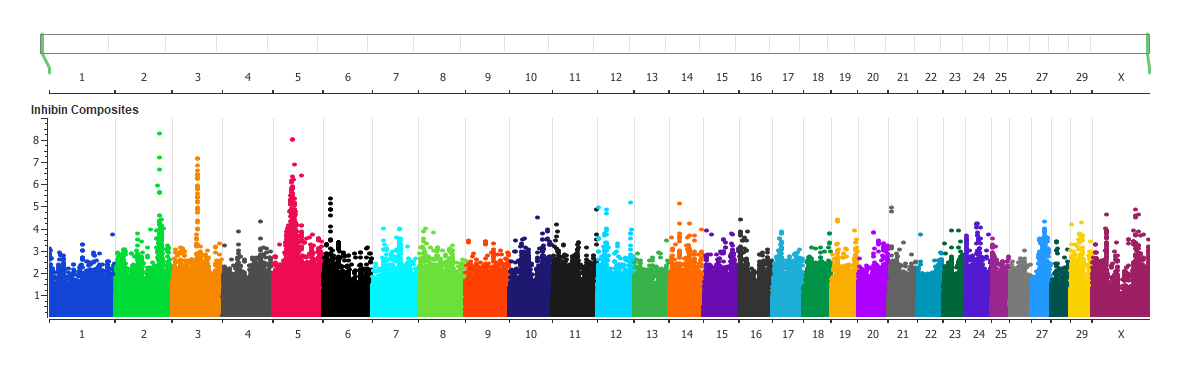
**

**Fig S5.** Manhattan plot for the analyses in each of the two breeds: SNP associations for Inhibin hormone levels (INH). Note that the SNP associations in chromosome 2 appear in both breeds, while associations in chromosome 3 and 5 only occurred in Tropical Composites.


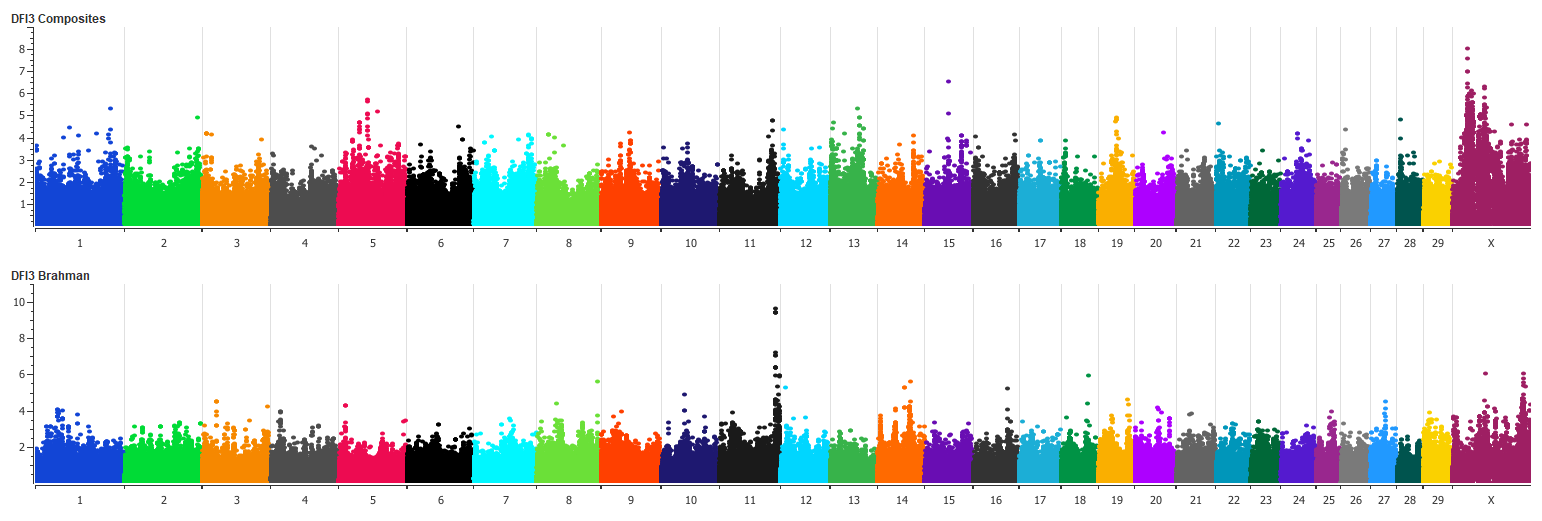


F**ig S6.** Manhattan plot for the analyses in each of the two breeds: SNP associations for the DNA fragmentation index (DFI3). Note that the association peak in chromosome 11 is only present in the Brahman analyses.

**
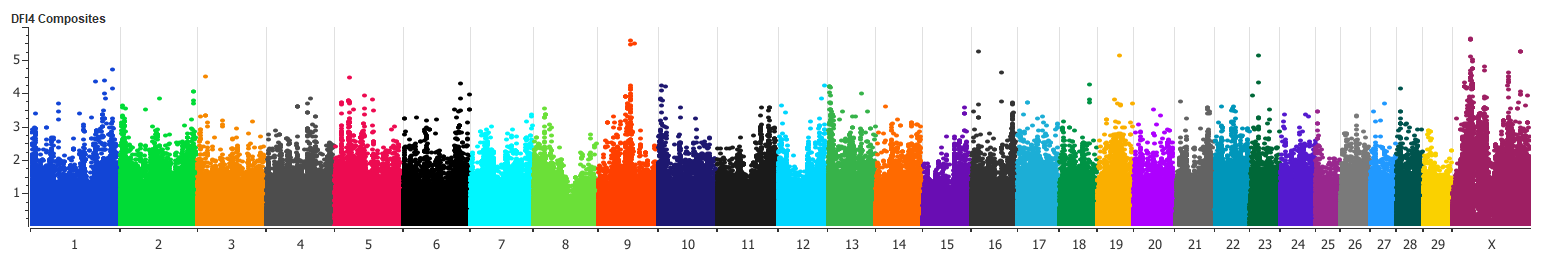

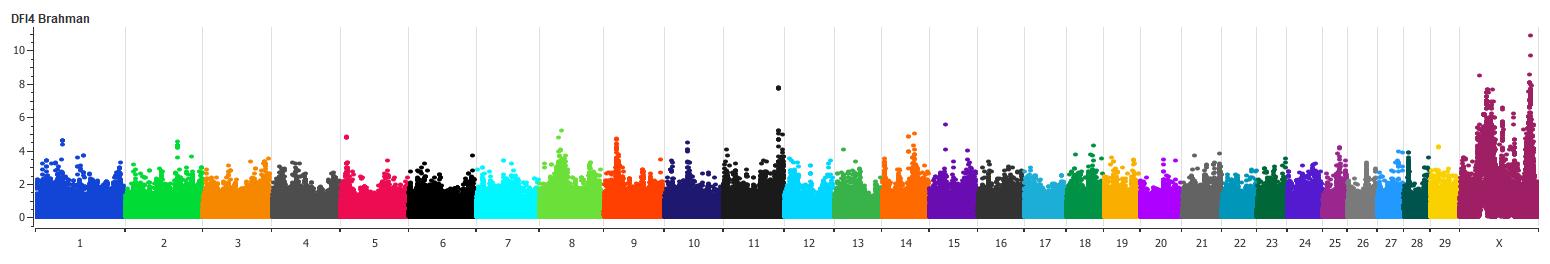
**

**Fig S7.** Manhattan plot for the analyses in each of the two breeds: SNP associations for the DNA fragmentation index measured with an alternative cytometry method (DFI4, see methods for phenotype details).

**
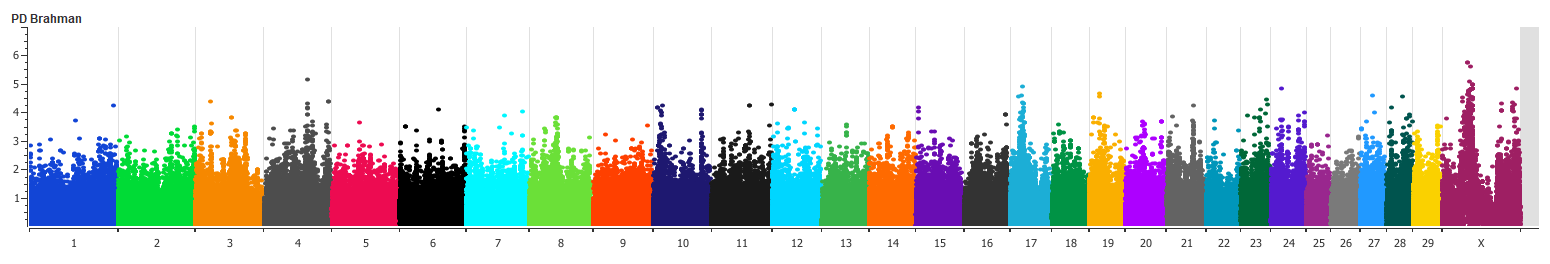

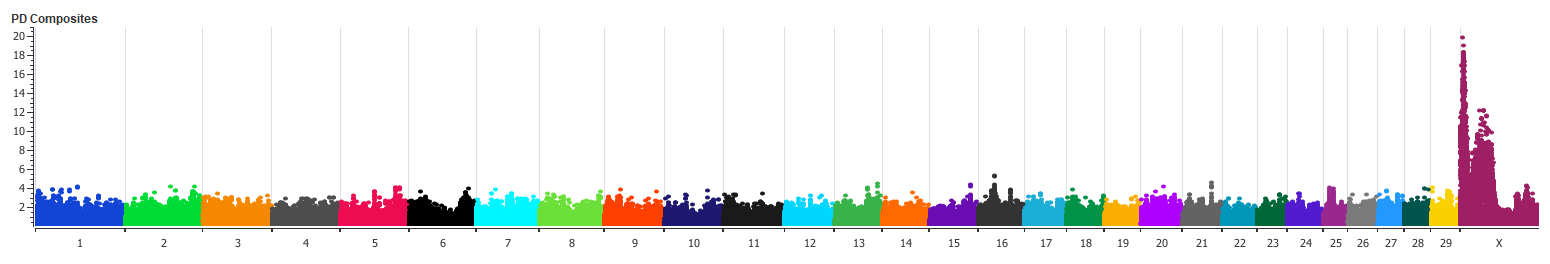
**

**Fig S8.** Manhattan plot for the analyses in each of the two breeds: SNP associations for the percentage of sperm with proximal droplets (PD). Note that the significant associations accumulate in the X chromsome and that there is evidence for more than one PD QTL in Tropical Composites. These PD associations seems to be breed specific, althought they overlap with the MA and the HA QTLs proposed from the Brahman results (Fig S3 and S4).

**
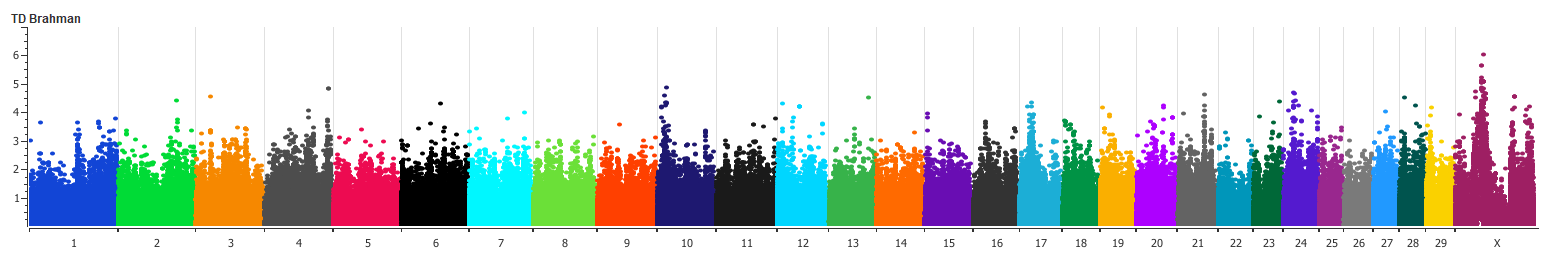

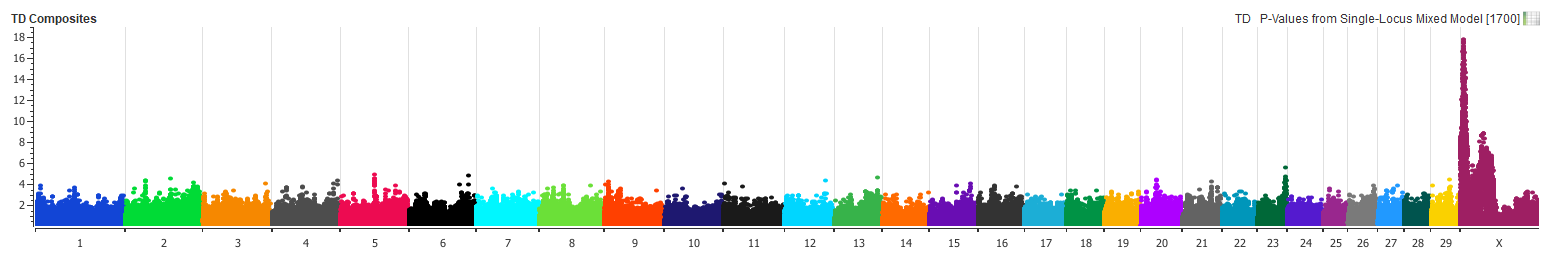
**

**Fig S9.** Manhattan plot for the analyses in each of the two breeds: SNP associations for the percentage of sperm with total droplets (TD). The total droplets SNP associations seem specific to Tropical Composites.

**
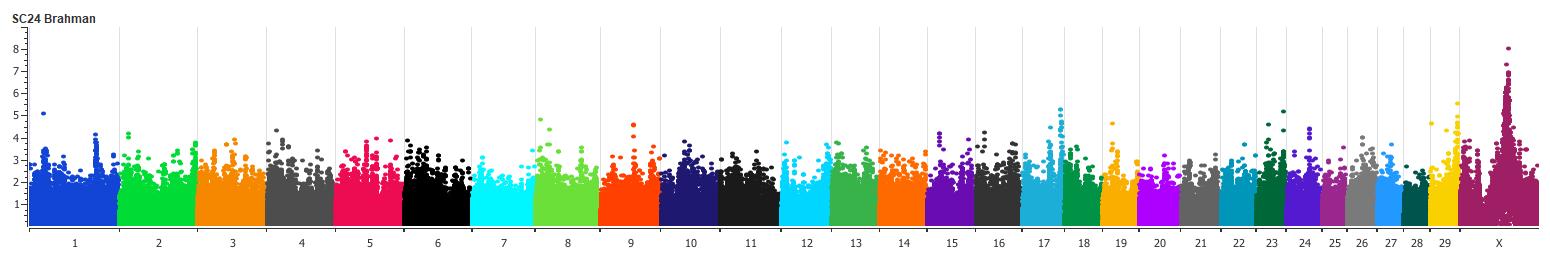
**

**
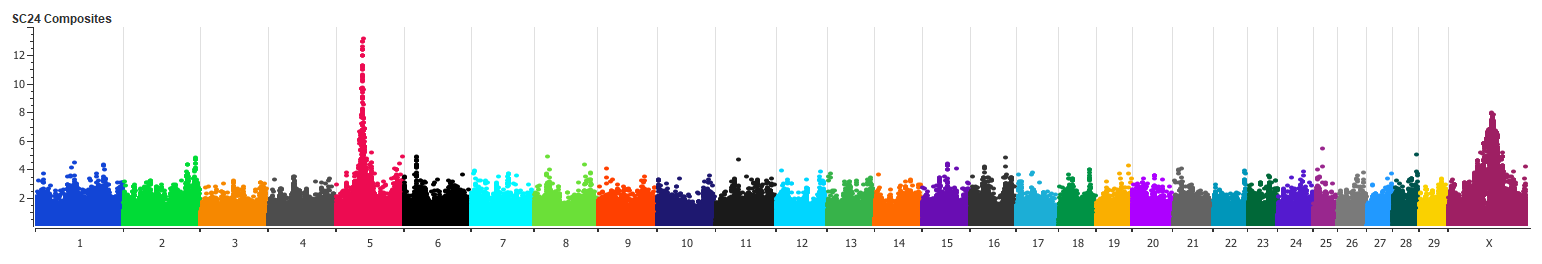
**

**Fig S10.** Manhattan plot for the analyses in each of the two breeds: SNP associations for scrotal circunference measured at approx 24 months of age (SC24). The significant associations observed in chromsome 5 led to proposing a QTL that is specific to Tropical Composites. SNP associations observed in both breeds suggest that the X chromosome could be important for scrotal circunference too.


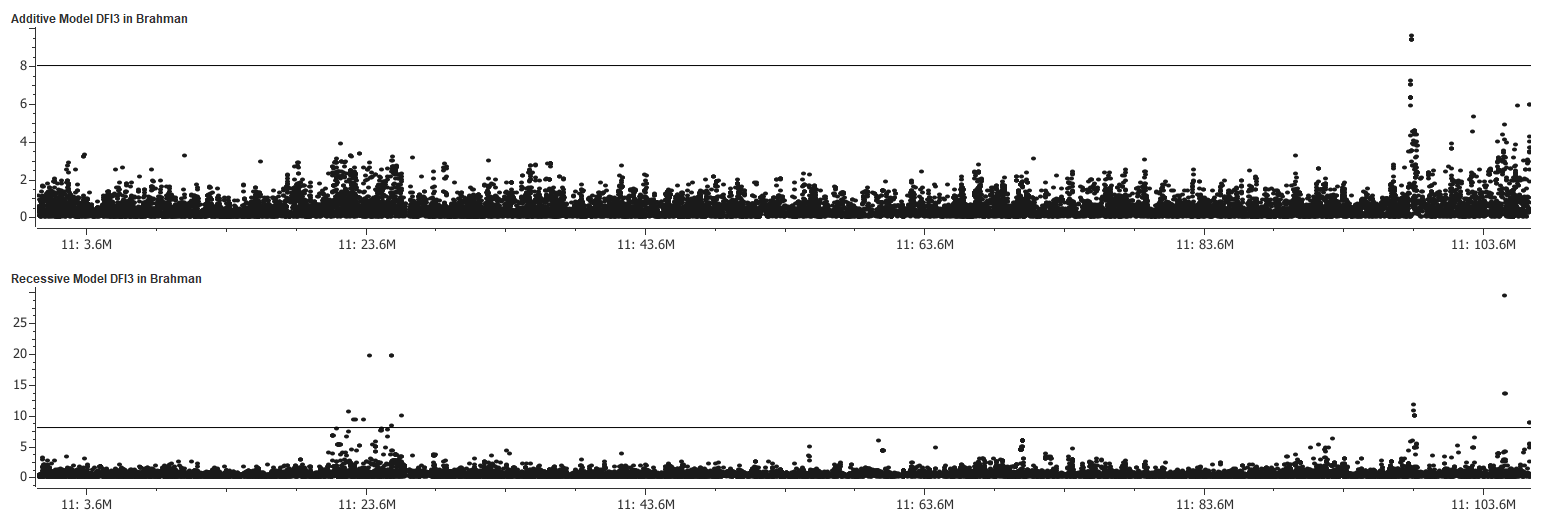


**Fig S11.** Manhattan plot for the additive (top) model and the recessive (bottom) model: SNP associations in chromsome 11 for DNA fragmentation index (DFI3) in Brahman.
